# Protein phosphatase 1 regulatory inhibitor subunit 14C promotes triple‐negative breast cancer progression via sustaining inactive glycogen synthase kinase 3 beta

**DOI:** 10.1002/ctm2.725

**Published:** 2022-01-28

**Authors:** Yunting Jian, Lingzhi Kong, Hongyi Xu, Yawei Shi, Xinjian Huang, Wenjing Zhong, Shumei Huang, Yue Li, Dongni Shi, Yunyun Xiao, Muwen Yang, Siqi Li, Xiangfu Chen, Ying Ouyang, Yameng Hu, Xin Chen, Libing Song, Runyi Ye, Weidong Wei

**Affiliations:** ^1^ Department of Experimental Research, Sun Yat‐sen University Cancer Center, State Key Laboratory of Oncology in South China Collaborative Innovation Center for Cancer Medicine Guangzhou China; ^2^ Department of Breast Surgery Sun Yat‐sen University Cancer Center Guangzhou China; ^3^ Department of Pathology, Key Laboratory of Reproduction and Genetics of Guangdong Higher Education Institutes, Key Laboratory for Major Obstetric Diseases of Guangdong Province The Third Affiliated Hospital of Guangzhou Medical University Guangzhou China; ^4^ Department of Thyroid and Breast Surgery The First Affiliated Hospital of Sun Yat‐sen University Guangzhou China; ^5^ Department of Biochemistry, Zhongshan School of Medicine Sun Yat‐sen University Guangzhou China; ^6^ Key Laboratory of Protein Modification and Degradation, School of Basic Medical Sciences; Guangzhou Institute of Oncology Tumor Hospital, Guangzhou Medical University Guangzhou China

**Keywords:** GSK3β, PPP1R14C, PRKCI, TNBC

## Abstract

Triple‐negative breast cancer (TNBC) is fast‐growing and highly metastatic with the poorest prognosis among the breast cancer subtypes. Inactivation of glycogen synthase kinase 3 beta (GSK3β) plays a vital role in the aggressiveness of TNBC; however, the underlying mechanism for sustained GSK3β inhibition remains largely unknown. Here, we find that protein phosphatase 1 regulatory inhibitor subunit 14C (PPP1R14C) is upregulated in TNBC and relevant to poor prognosis in patients. Overexpression of PPP1R14C facilitates cell proliferation and the aggressive phenotype of TNBC cells, whereas the depletion of PPP1R14C elicits opposite effects. Moreover, PPP1R14C is phosphorylated and activated by protein kinase C iota (PRKCI) at Thr73. p‐PPP1R14C then represses Ser/Thr protein phosphatase type 1 (PP1) to retain GSK3β phosphorylation at high levels. Furthermore, p‐PPP1R14C recruits E3 ligase, TRIM25, toward the ubiquitylation and degradation of non‐phosphorylated GSK3β. Importantly, the blockade of PPP1R14C phosphorylation inhibits xenograft tumorigenesis and lung metastasis of TNBC cells. These findings provide a novel mechanism for sustained GSK3β inactivation in TNBC and suggest that PPP1R14C might be a potential therapeutic target.

## Graphical abstract

- PPP1R14C is upregulated in TNBC, and promotes tumorigenesis and metastasis.
- PPP1R14C inhibits GSK3β by retaining its Ser9 phosphorylation and inducing its protein degradation.
- PPP1R14C is phosphorylated and activated by PRKCI.
- Inhibition of PPP1R14C phosphorylation represents a promising therapeutic strategy against TNBC

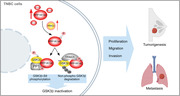

## 1 BACKGROUND

Triple‐negative breast cancer (TNBC) is an aggressive breast cancer subtype, which lacks the expression of estrogen receptor (ER), progesterone receptor (PR), and human epidermal growth factor receptor‐2 (HER‐2).^1^ Given less benefit from endocrine therapy or anti‐HER‐2 therapy, traditional chemotherapy is identified as the optimal strategy for TNBC.^2^, ^3^ However, the response of available chemotherapies is often short‐term as the tumour progresses and acquires resistance.^4^ Hence, it is imperative to develop innovative and effective treatments for TNBC, especially treatments against potential targets.

Glycogen synthase kinase 3 beta (GSK3β), a highly evolutionarily conserved Ser/Thr kinase, is engaged in the cancer development and progression through its selective phosphorylation of substrate proteins.^5^, ^6^ GSK3β is active and acts as a tumour suppressor by phosphorylating and destabilizing oncogenic transcription factors (TFs).^8^ On the other hand, in malignancy, GSK3β is phosphorylated at residue Ser9 into an inactive state, resulting in the activation of downstream oncogenic signalling.^9^, ^10^, ^11^, ^12^, ^13^ It has been reported that the elevation of p‐GSK3β‐Ser9 accumulates snail family transcriptional repressor (SLUG) and snail family transcriptional repressor 1 (SNAIL) proteins, and expedites epithelial–mesenchymal transition (EMT) and metastasis in non‐small cell lung carcinoma cells.^14^ Increased p‐GSK3β‐Ser9 promotes cell proliferation and tumour growth in TNBC. Ser/Thr protein phosphatase type 1 (PP1), one of the phosphatases, is identified as a major regulator of the homeostasis of GSK3β status.^16^ Notably, GSK3β is reported to stay inactive in TNBC and other malignancies, indicating the roles of phosphatase might be deregulated in cancers^17^, ^18^, ^19^; however, the mechanisms remain unclear.

*PPP1R14C*, found as a morphine‐regulated brain gene, encodes protein phosphatase 1 regulatory inhibitor subunit 14C, and is identified as a potent inhibitor of PP1.^20^, ^21^ Horvath and colleagues reported that PPP1R14C inhibited the release of neurotransmitters and promoted neuronal exocytosis by retaining synaptosome associated protein 25 (SNAP25) at a phosphorylated state.^22^ Moreover, overexpression of PPP1R14C increased the phosphorylation of RB transcriptional co‐repressor 1 (RB1) to protect leukemic cells from chemotherapy.^23^ These findings suggest that PPP1R14C might be involved in human cancer progression by regulating the phosphorylation state of a particular protein. However, the role of PPP1R14C in TNBC remains unknown.

In the present study, we find that PPP1R14C is robustly increased in TNBC and predicts poor prognosis in patients. PPP1R14C is phosphorylated and activated by protein kinase C iota (PRKCI) at Thr73. p‐PPP1R14C then binds with PP1 and inhibits its phosphatase activity to increase inactive p‐GSK3β‐Ser9. p‐PPP1R14C recruits E3 ligase TRIM25 to promote the ubiquitylation and degradation of non‐phosphorylated GSK3β. Thus, PPP1R14C promotes the aggressiveness, tumour growth, and metastasis of TNBC cells in vitro and in vivo by sustaining inactive GSK3β. Importantly, inhibition of PPP1R14C phosphorylation showed the anti‐cancer activity. These findings uncover an oncogenic role of PPP1R14C in TNBC, and suggest that PPP1R14C might be a potential marker or target.

## 2 RESULTS

### 2.1 PPP1R14C is specifically upregulated in TNBC

**FIGURE 1:**
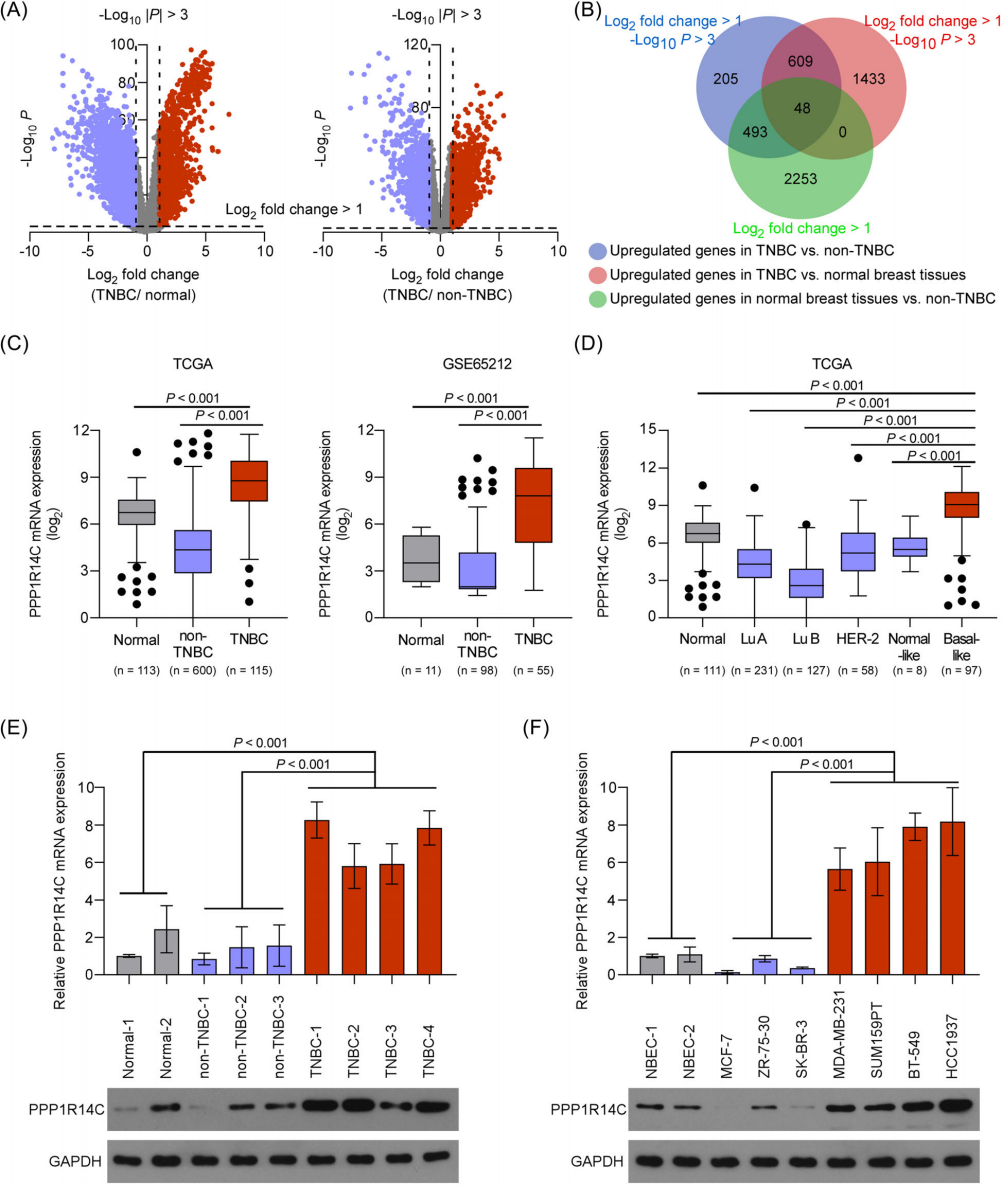
PPP1R14C is specifically upregulated in triple‐negative breast cancer (TNBC). (A) Volcano plots showing gene expression in The Cancer Genome Atlas (TCGA) breast cancer dataset comparing TNBC with normal tissues (left panel), and comparing TNBC with non‐TNBC (right panel). The blue dots represent the downregulated genes and red dots represent the upregulated genes. (B) A Venn diagram of the upregulated genes in TNBC compared with non‐TNBC, TNBC compared to normal samples, and normal tissues compared to non‐TNBC. (C) PPP1R14C mRNA expression levels in TCGA breast cancer dataset (including 113 normal, 115 TNBC, and 600 non‐TNBC samples) and a Gene expression Omnibus (GEO) dataset (GSE65212, including 11 normal, 55 TNBC, and 98 non‐TNBC samples). (D) PPP1R14C mRNA expression levels in tumour samples with informed molecular subtypes from the TCGA breast cancer dataset. Statistical analysis comprised normalization to the expression levels in the basal‐like subgroup. Lu A, luminal A; Lu B, luminal B. (E and F) Quantitative real‐time PCR (qRT‐PCR) (up) and western blotting (down) analyses of PPP1R14C expression in human breast cancer tissues (E) and cell lines (F). GAPDH was used as a loading control. Data represent the means ± S.D. of three independent experiments.

Identifying genes specifically deregulated in TNBC might provide potential targetable molecular vulnerabilities for this intractable breast cancer subtype. Here, we first analysed The Cancer Genome Atlas (TCGA) breast cancer dataset. We identified 48 genes that were significantly increased in TNBC, by at least two folds compared to that in normal and non‐TNBC tissues (Figure 1A,B). Among the 48 genes, some of them, such as FOXC1,^24^ BCL11A,^25^ and PSAT1,^26^ have already been reported to play important roles in TNBC (Table S1), suggesting that the analysis was reliable. Notably, PPP1R14C was previously identified as an inhibitor of PP1 and found to decrease the chemosensitivity of leukemic cells.^20^, ^23^ This prompted us to further explore the prospective role of PPP1R14C in TNBC progression.

The specific upregulation of PPP1R14C could also be observed in Gene expression Omnibus (GEO) dataset as shown by the TCGA dataset (Figure 1C). Meanwhile, PPP1R14C was highly expressed in basal‐like breast cancer (BLBC), followed by normal tissues and other subtypes (Luminal A, Luminal B, and HER‐2) (Figure 1D). Importantly, our real‐time PCR and western blotting analyses did show that PPP1R14C was robustly upregulated in TNBC tumour tissues and cell lines (Figure 1E,F). These results indicated a specific upregulation of PPP1R14C in TNBC.

### 2.2 High PPP1R14C expression indicates a poor prognosis in patients with TNBC

**FIGURE 2:**
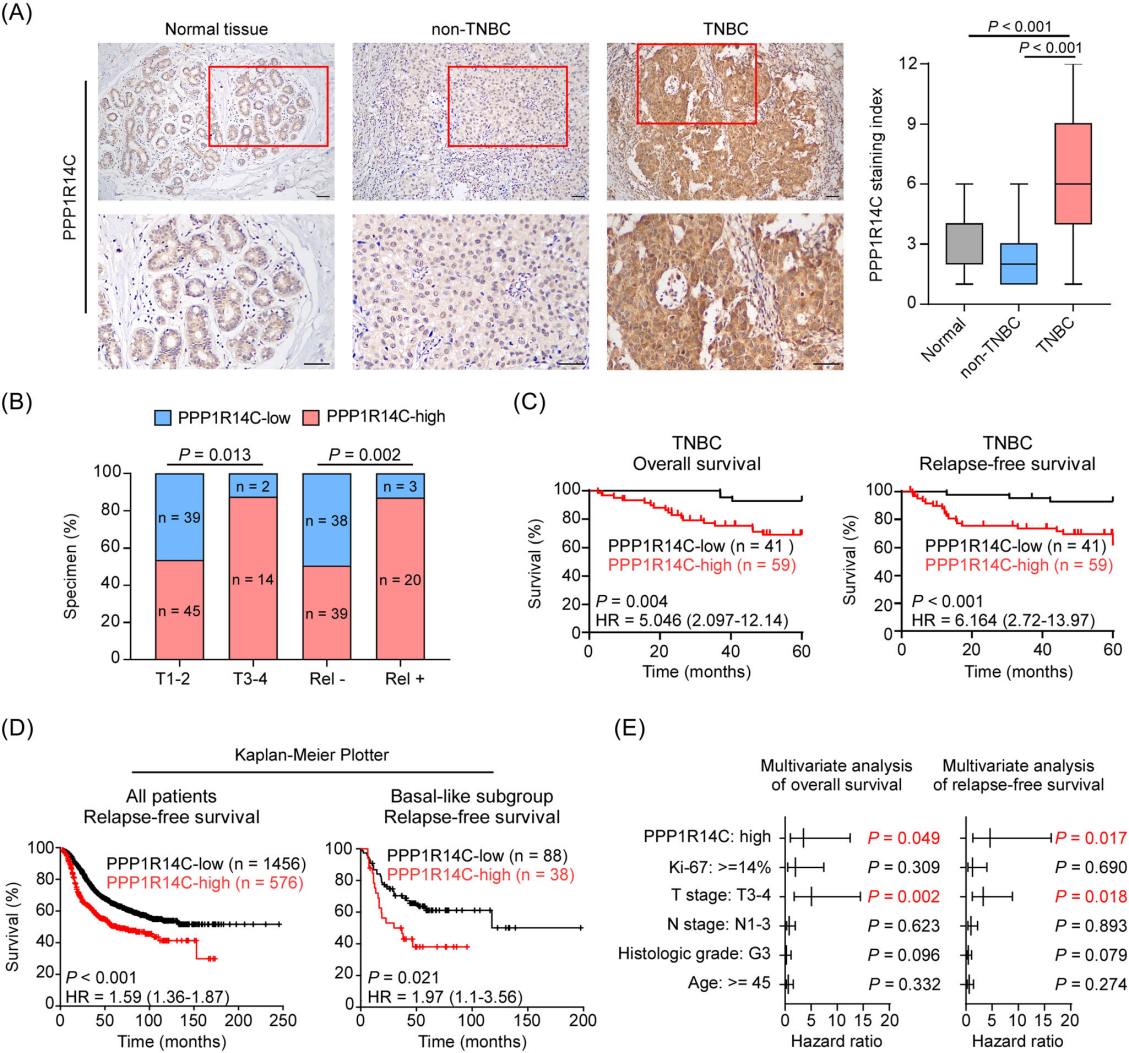
Upregulation of PPP1R14C is associated with poor prognosis in patients with triple‐negative breast cancer (TNBC). (A) Representative images of PPP1R14C staining in normal breast tissues, non‐TNBC, and TNBC tissues (left panel). The staining index (SI) distribution of each group was indicated (right panel) and the Mann–Whitney *U*‐test was used for analysis. Scale bars represent 50 μm. (B) The distribution and correlation between PPP1R14C staining and T stage, and relapse status in patients with TNBC. The χ^2^ test was used for analysis. Rel−, no relapse; Rel +, with relapse. (C) Kaplan–Meier 5‐year overall survival (OS) and relapse‐free survival (RFS) curve for patients with TNBC stratified by low (*n* = 41) and high PPP1R14C expression (*n* = 59, log‐rank test). HR, hazard ratio. (D) The Kaplan–Meier Plotter (http://kmplot.com/analysis) program was used to analyse relapse‐free survival of all patients with breast cancer and the basal‐like subgroup (Pietenpol subtype). All settings were left at default values except for: gene symbol (PPP1R14C), survival (RFS), and auto‐select best cutoff (on). (E) Multivariate Cox regression analysis to evaluate the significance of the association between PPP1R14C signature and OS, and RFS in the presence of other clinical variables.

The expression and clinical significance of PPP1R14C were then assessed by immunohistochemistry (IHC) and survival analysis in 150 breast cancer patient specimens, including 50 non‐TNBCs and 100 TNBCs (Supplementary Table 2). Consistently, the IHC analysis indicated strong staining of PPP1R14C in TNBC, but it was weakly expressed in normal breast tissues and non‐TNBC tissues (Figure 2A). The staining of PPP1R14C expression was evaluated by the staining index (SI) according to the intensity and density, and an SI ≥ 6 was defined as PPP1R14C‐high. High PPP1R14C expression was positively correlated with advanced T stage (*P* = 0.013), and relapse status (*P* = 0.002) in patients with TNBC (Figure 2B and Table S3). Importantly, TNBC patients with high PPP1R14C expression experienced poor overall survival (OS) and relapse‐free survival (RFS; Kaplan–Meier survival curves and log‐rank test; *P* = 0.004, hazard ratio (HR) = 5.046, 95% confidence interval (CI) = 2.097–12.14; *P* < 0.001, HR = 6.164, 95% CI = 2.72–13.97, respectively; Figure 2C, Table S4). In addition, using the online database Kaplan–Meier plotter (http://kmplot.com/analysis), we found that patients with high PPP1R14C expression suffered shorter RFS among all patients with breast cancer and basal‐like subgroup (Figure 2D). High PPP1R14C expression and advanced T stage were identified as independent prognostic factors for five‐year OS and RFS in TNBC by multivariate regression analysis (Figure 2E and Table S4). These results suggested that upregulation of PPP1R14C might contribute to the malignant progression of TNBC.

### 2.3 PPP1R14C promotes TNBC progression in vitro

**FIGURE 3:**
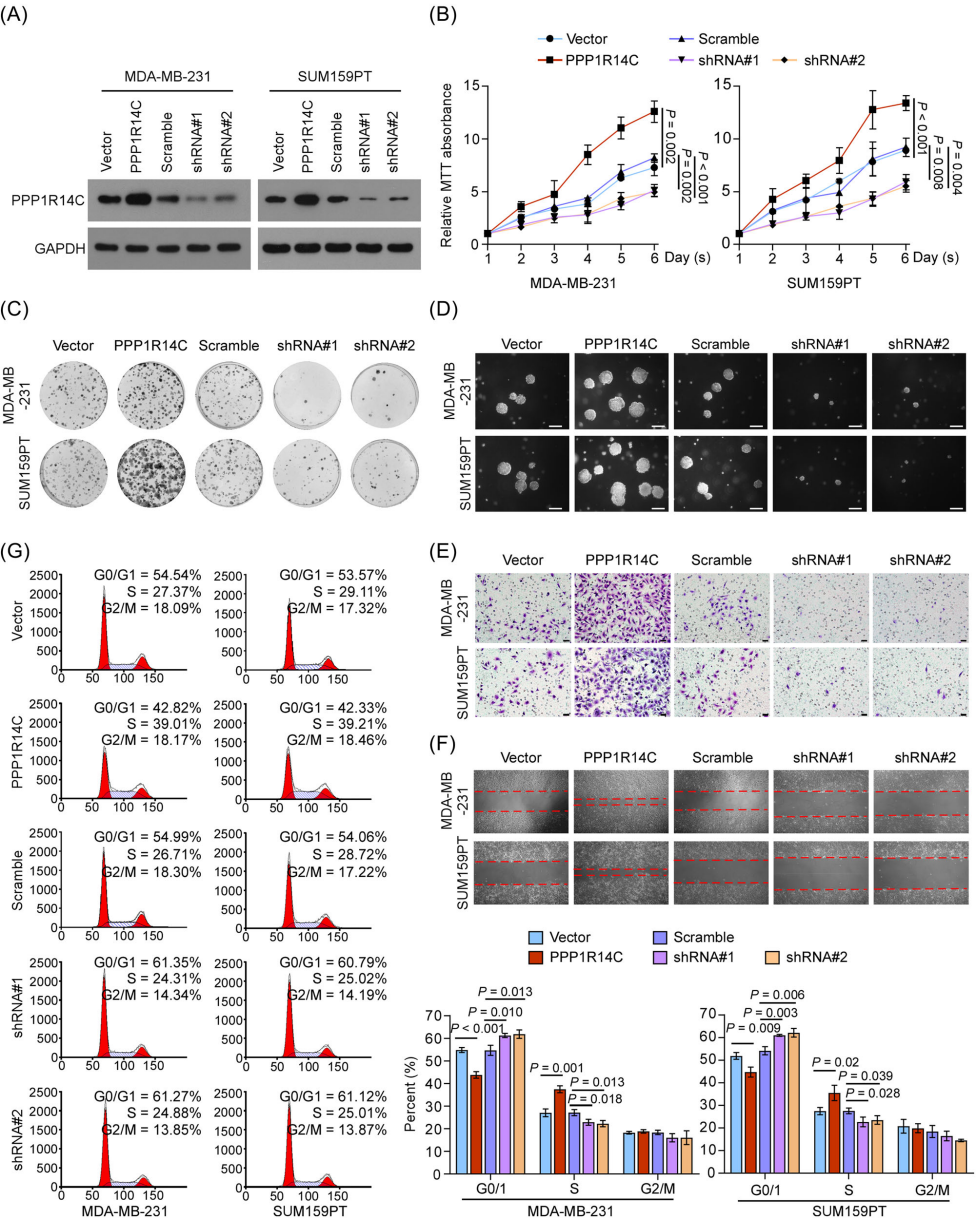
PPP1R14C promotes triple‐negative breast cancer (TNBC) progression in vitro. (A) Western blotting analysis of PPP1R14C in MDA‐MB‐231 and SUM159PT cells stably transduced with PPP1R14C‐overexpressing and PPP1R14C‐silencing plasmids. GAPDH was used as a loading control. (B–G) MTT (B), colony formation (C), anchorage‐independent growth (D), transwell (E), wound healing (F) assays, and flow cytometric analysis (G) were performed in the indicated cells. Scale bars represent 50 μm. A two‐tailed Student's *t*‐test was used. Data represent the means ± S.D. of three independent experiments.

The roles of PPP1R14C in TNBC progression were then investigated by gain or loss‐of‐function methods. PPP1R14C was exogenously transduced or endogenously silenced in two human breast cancer cell lines (MDA‐MB‐231 and SUM159PT) (Figure 3A). Significantly, overexpression of PPP1R14C promoted cell proliferation, colony formation, anchorage‐independent growth, invasion, migration, and cell cycle transition in TNBC cells (Figure 3B–G and Figure S1A–D). In contrast, depletion of PPP1R14C had opposite effects (Figure 3B–G and Figure S1A–D). Moreover, similar tumour‐promoting effects were also observed when using a mouse‐derived TNBC cell line 4T1, suggesting that the role of PPP1R14C was conserved (Figure S1E–J). These results showed that PPP1R14C promoted TNBC progression in vitro.

### 2.4 PPP1R14C facilitates TNBC tumour growth and metastasis

**FIGURE 4:**
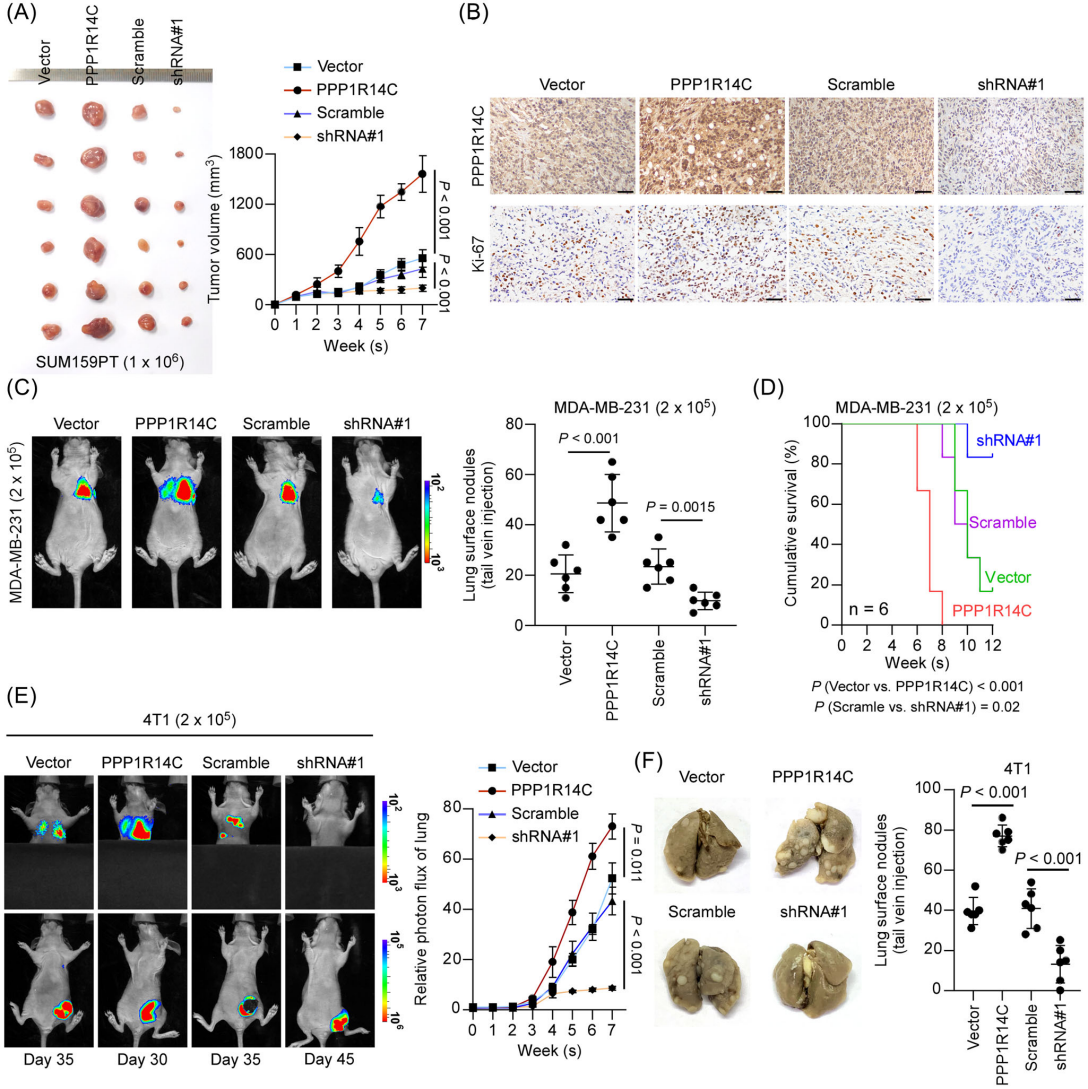
PPP1R14C facilitates triple‐negative breast cancer (TNBC) tumour growth and metastasis. (A) Control, PPP1R14C‐overexpressing, or PPP1R14C‐silenced SUM159PT cells were orthotopically injected into mice (1 × 10^6^/injection, *n* = 6/group). The tumour volumes in each group were shown. (B) IHC of Ki‐67 staining showed in the indicated xenografts. Scale bars represent 50 μm. (C) In vivo metastasis assays of PPP1R14C‐overexpressing, PPP1R14C‐silenced, and control MDA‐MB‐231 cells. Lung metastatic burden was monitored weekly using bioluminescent imaging (BLI). Representative BLI images of the lungs were shown. The visible surface metastatic lesions were counted. (D) Kaplan–Meier survival curves of mice. (E) Lung colonization and spontaneous metastasis models of PPP1R14C‐overexpressing, PPP1R14C‐silenced, and control 4T1 cells. BLI quantification of lung metastasis of the indicated cells. (F) The visible surface metastatic lesions of the lungs were counted. Two‐tailed Student's *t*‐test and log‐rank test were used.

The effects of PPP1R14C on TNBC progression were further assessed in vivo. Xenograft mice model was generated through the orthotopic injection of stable SUM159PT cell lines and tumour burdens were measured regularly. Compared with the control groups, tumour growth was remarkably accelerated in the PPP1R14C‐overexpressing group, or suppressed in the PPP1R14C‐silenced group (Figure 4A and Figure S2A). Tumours with overexpression of PPP1R14C had a high level of Ki‐67, whereas the PPP1R14C‐silenced tumours showed decreased Ki‐67 levels (Figure 4B and Figure S2B). Moreover, the impacts of PPP1R14C on TNBC metastasis were determined in the lung colonization model. Luciferase‐transduced MDA‐MB‐231 cell lines were injected into the tail veins of mice and metastatic burdens were monitored by bioluminescence imaging (BLI) weekly. Upregulation of PPP1R14C increased the lung metastatic burdens and deteriorated the survival of mice, while downregulation of PPP1R14C reduced the number of lung metastatic lesions and prolonged mice survival (Figure 4C,D).

Furthermore, the role of PPP1R14C in metastasis was explored in an orthotopic mouse model of spontaneous breast cancer metastasis using the 4T1 cells. Luciferase‐expressing 4T1 cells (2 × 10^5^) with altered PPP1R14C expression were orthotopically injected into the mammary fat pad. Tumour volumes were determined every week after surgery. The spontaneous metastasis of 4T1 cells was evaluated using the Xenogen IVIS Spectrum Imaging System. Strikingly, BLI and the visible metastatic lesions revealed that the metastasis of 4T1 cells was promoted in the PPP1R14C overexpression group, but suppressed in the PPP1R14C‐silenced group (Figure 4E,F). Moreover, upregulating PPP1R14C expression shortened the survival time of mice strikingly (Figure S2C). These findings indicated that PPP1R14C promoted tumorigenesis and metastasis of TNBC.

### 2.5 PPP1R14C inactivates PP1 to increase phosphorylation of GSK3β

**FIGURE 5:**
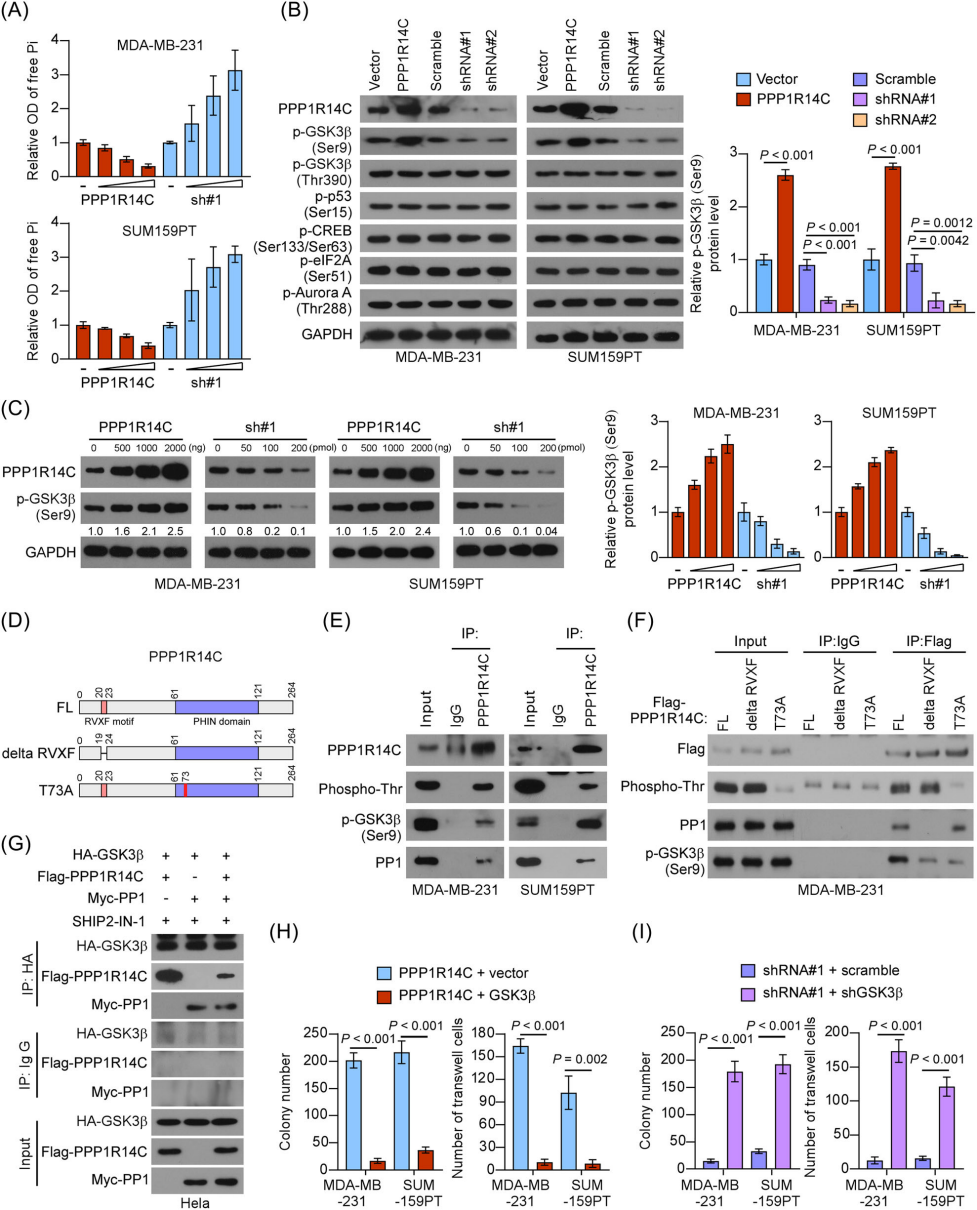
PPP1R14C inactivates PP1 to increase GSK3β phosphorylation. (A) The phosphatase activity of PP1 was measured by detecting its product, free Pi, in the triple‐negative breast cancer (TNBC) cells transfected with increasing dose of PPP1R14C‐expressing (0 ng, 500 ng, 1000 ng, and 2000 ng) or PPP1R14C‐shRNA (0 pmol, 50 pmol, 100 pmol, and 200 pmol) plasmids. (B) Left panel: western blotting analysis of PPP1R14C, p‐GSK3β (Ser9), p‐GSK3β (Thr390), p‐p53 (Ser15), p‐CREB (Ser133/Ser63), p‐eIF2A (Ser51), and p‐Aurora A (Thr288) in PPP1R14C‐transduced cells and PPP1R14C‐silenced cells compared with control cells. GAPDH was used as a loading control. Right panel: the statistical graph of protein level of p‐GSK3β (Ser9). Immunoblots were representative of three biological replicates. (C) Left panel: the expression of p‐GSK3β (Ser9) was detected by western blotting assay in the TNBC cell lines transfected with increasing dose of PPP1R14C‐overexpressing (0 ng, 500 ng, 1000 ng, and 2000 ng) or PPP1R14C‐shRNA (0 pmol, 50 pmol, 100 pmol, and 200 pmol) plasmids. GAPDH was used as a loading control. Right panel: the statistical graph of protein level of p‐GSK3β (Ser9). Immunoblots were representative of three biological replicates. (D) The truncated mutants of PPP1R14C were based on known functional domains. RVXF motif: Arg/Lys–Val/Ile–Xaa–Phe motif; PHIN domain: PP1 Holoenzyme Inhibitory domain. (E) IP assays revealed the interaction of PPP1R14C, p‐PPP1R14C, PP1, and p‐GSK3β (Ser9) in SUM159PT and MDA‐MB‐231 cells. (F) Detailed interactions between PPP1R14C, PP1, and p‐GSK3β (Ser9) were analysed by IP assays. (G) Extraneous IP assays detecting the interaction of PPP1R14C‐PP1 with p‐GSK3β (Ser9) under SHIP2‐IN‐1 treatment (10 μM, 24 h). (H) Colony formation and transwell assays were performed in PPP1R14C + vector, PPP1R14C + GSK3β cell lines. (I) Colony formation and transwell assays were performed in shPPP1R14C#1 + scramble, shPPP1R14C#1 + shGSK3β cells. A two‐tailed Student's *t*‐test was used.

We next explored the mechanism for PPP1R14C in TNBC. PPP1R14C was identified as a potent inhibitor of PP1.^27^ Indeed, our results showed that upregulation of PPP1R14C reduced, while silencing PPP1R14C increased the activity of PP1 in TNBC cells (Figure 5A). Interestingly, overexpressing PPP1R14C upregulated, while silencing PPP1R14C reduced the Ser9 phosphorylation level of GSK3β, but did not influence the other substrate proteins^28^, ^29^, ^30^, ^31^, ^32^, ^33^ (Figure 5B). PPP1R14C overexpression increased the level of p‐GSK3β‐Ser9, while silencing PPP1R14C showed the opposite effect, in a dose‐dependent manner (Figure 5C). Moreover, PPP1R14C overexpression decreased, while knockdown of PPP1R14C increased the expression levels of GSK3β downstream proteins (Figure S3A). These findings indicated that PPP1R14C substantially promoted GSK3β phosphorylation at Ser9.

Previous studies revealed that the RVXF motif and T73 phosphorylation were required for PPP1R14C to bind and inhibit PP1, respectively^34^, ^35^, ^36^ (Figure 5D). IP assays using antibodies against PPP1R14C demonstrated that p‐PPP1R14C/PP1/p‐GSK3β‐Ser9 formed a complex in TNBC cells (Figure 5E). Next, we used two PPP1R14C mutants that one was no longer bound to PP1 (deletion of the RVXF motif) and the other was not inhibitory to PP1 (T73A) to perform IP assays. The results showed that neither of the PPP1R14C mutants could restore the p‐GSK3β‐Ser9 level in TNBC cells (Figure 5F). Moreover, cell lysates obtained from Hela cells transduced with HA‐GSK3β, Flag‐PPP1R14C, and Myc‐PP1 were detected using IP assays under treatment with SHIP2‐IN‐1, a GSK3β inhibitor that deactivates GSK3β via phosphorylation at Ser9.^37^ The assays showed that PPP1R14C and PP1 specifically interacted with p‐GSK3β (Figure 5G). These results suggested that PPP1R14C inactivated GSK3β by sustaining its phosphorylation.

We further investigated whether inhibition of GSK3β was essential for the tumour‐promoting functions of PPP1R14C in TNBC. As expected, ectopic expression of GSK3β substantially impaired the colony formation, invasion, and migration capacities of TNBC cells with upregulated PPP1R14C (Figure 5H and Figure S3B). In contrast, depletion of GSK3β rescued the aggressiveness in PPP1R14C‐silencing TNBC cells (Figure 5I and Figure S3C). The above data indicated that GSK3β was indeed a potent downstream effector of PPP1R14C.

### 2.6 PRKCI is an upstream regulator of PPP1R14C

**FIGURE 6:**
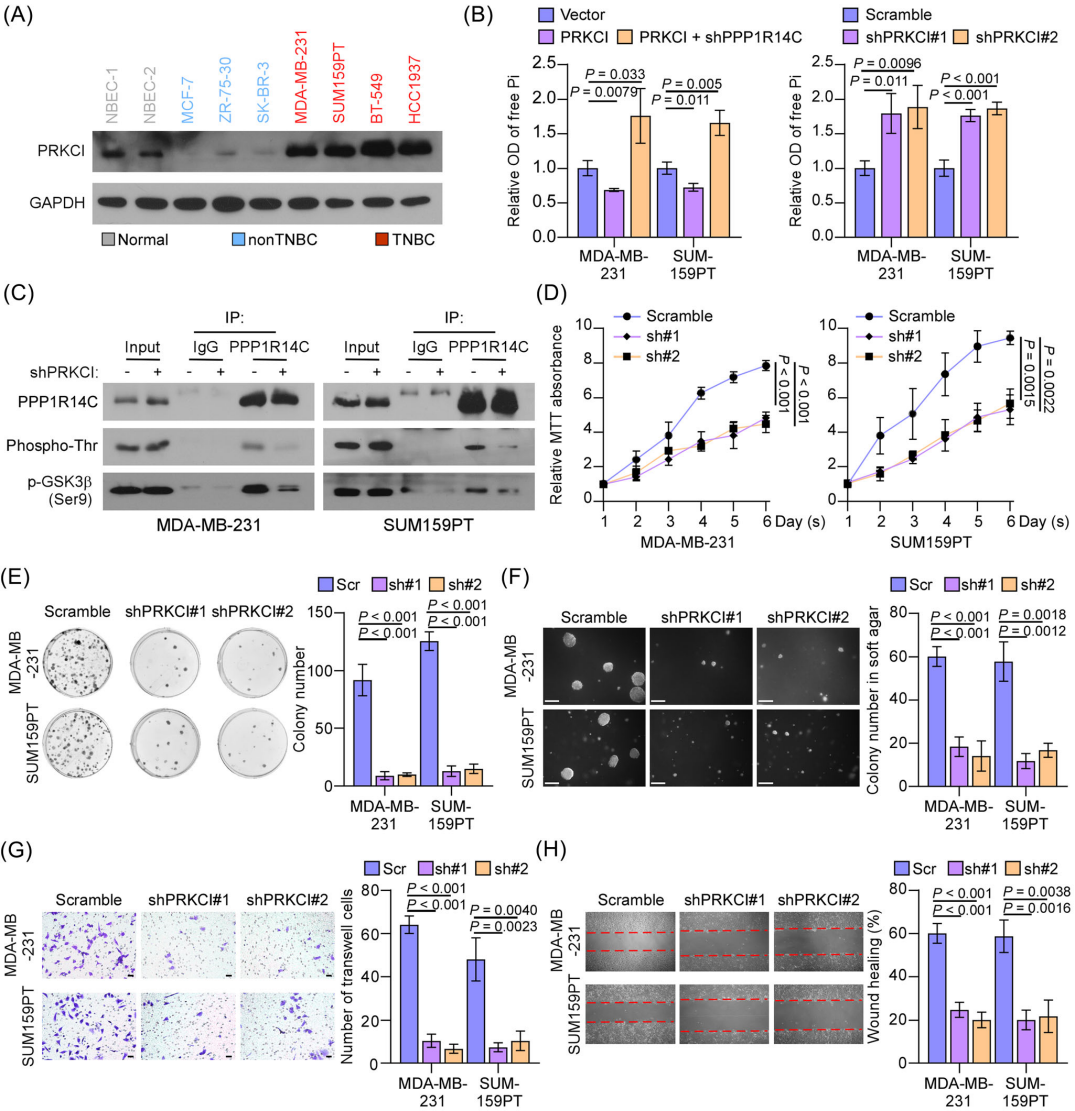
PRKCI is an upstream regulator of PPP1R14C. (A) Western blotting analysis of PRKCI expression in human breast cancer cell lines. GAPDH was used as a loading control. (B) The phosphatase activity of PP1 was measured by detecting its product, free Pi, in the indicated triple‐negative breast cancer (TNBC) cells. (C) TNBC cells transfected with or without shPRKCI were subjected to IP assays with an anti‐PPP1R14C antibody followed by western blotting with anti‐Phospho‐Thr and anti‐p‐GSK3β (Ser9) antibodies. (D–H) MTT (D), colony formation (E), anchorage‐independent growth (F), transwell (G), and wound healing (H) assays were performed in scramble, shPRKCI#1, and shPRKCI#2 cell lines. A two‐tailed Student's *t*‐test was used. Immunoblots were representative of three biological replicates.

Previous studies showed that phosphorylation of PPP1R14C was mainly dependent on protein kinase C (PKC).^20^ To identify which PKC family member might regulate PPP1R14C in TNBC, we analysed PKC genes expression in the TCGA breast cancer dataset. Notably, we found that, among the PKC members, PRKCI was expressed abundantly and significantly increased in TNBC compared to normal tissues and non‐TNBC (Figure S4A). Strikingly, our western blotting analysis showed that PRKCI was specifically upregulated in TNBC cell lines (Figure 6A), suggesting that PRKCI might be the select PKC for PPP1R14C activation. Indeed, the PP1 activity was reduced in PRKCI‐overexpressing cells and could be rescued by silencing PPP1R14C. In contrast, silencing PRKCI significantly increased PP1 activities in TNBC cells (Figure 6B). Subsequently, depletion of PRKCI remarkably reduced Thr73 phosphorylation of PPP1R14C and Ser9 phosphorylation of GSK3β (Figure 6C). These findings indicated that PRKCI regulated the phosphorylation and activity of PPP1R14C in TNBC. Nevertheless, we speculated that there might be other kinase contributing to the phosphorylation of PPP1R14C, which remained to be investigated in the future.

Notably, similar to PPP1R14C depletion, silencing PRKCI suppressed cell proliferation, colony formation, anchorage‐independent growth, invasion, migration, and cell‐cycle transition in human and mouse TNBC cells (Figure 6D–H and Figure S4B–E), suggesting that PRKCI, being an upstream activator of PPP1R14C, did play a tumour‐promoting role in TNBC progression.

### 2.7 p‐PPP1R14C facilitates non‐phosphorylated GSK3β (S9A) degradation

**FIGURE 7:**
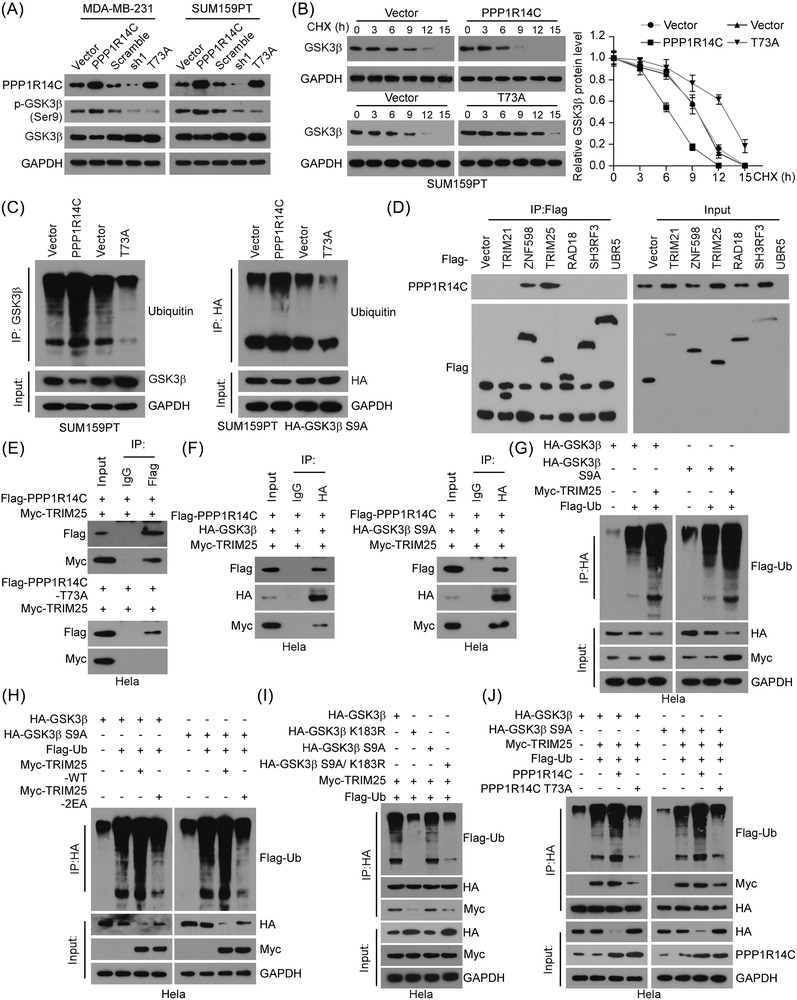
p‐PPP1R14C facilitates non‐phosphorylated glycogen synthase kinase 3 beta (GSK3β) (S9A) degradation. (A) Western blotting analysis of PPP1R14C, p‐GSK3β (Ser9), and GSK3β in PPP1R14C‐overexpressed, PPP1R14C‐silenced or PPP1R14C (T73A)‐transduced cells compared with control cells. GAPDH was used as a loading control. (B) Left panel: western blotting analysis of GSK3β protein in the indicated SUM159PT cells treated with CHX (50 μg/ml) for 0, 30, 60, or 120 min. GAPDH was used as a loading control. Right panel: the statistical graph of protein level of GSK3β. Immunoblots were representative of three biological replicates. (C) Effect of ubiquitination on GSK3β and non‐phosphorylated GSK3β (S9A) by IP assays in SUM159PT or SUM159PT‐HA‐GSK3β (S9A) cells using anti‐GSK3β or anti‐HA antibodies to pull‐down, respectively. (D) Hela cells transfected with Flag‐vector, ‐TRM21, ‐ZNF598, ‐TRIM25, ‐RAD18, ‐SH3RF3, and ‐UBR5 were subjected to IP assays with an anti‐Flag antibody, followed by western blotting with an anti‐PPP1R14C antibody. (E) IP assays revealed the interaction between Flag‐PPP1R14C or ‐PPP1R14C (T73A), and Myc‐TRIM25 in Hela cells. (F) IP assays revealed the interaction between Flag‐PPP1R14C, Myc‐TRIM25, and GSK3β/non‐phosphorylated GSK3β (S9A) in Hela cells. (G) Hela cells transfected with HA‐GSK3β, non‐phosphorylated HA‐GSK3β (S9A), and Flag‐Ub along with Myc‐TRIM25 plasmids were subjected to IP assays with an anti‐HA antibody, followed by western blotting with anti‐Flag antibody. (H) Hela cells transfected with Flag‐Ub, Myc‐TRIM25 or Myc‐TRIM25 (2EA) along with HA‐GSK3β or HA‐GSK3β (S9A) were subjected to IP assays with an anti‐HA antibody, followed by western blotting with an anti‐Flag antibody. (I) Hela cells transfected with HA‐GSK3β, HA‐GSK3β (K183R, S9A, or S9A/K183R), along with Myc‐TRIM25 and Flag‐Ub were subjected to IP assays with an anti‐HA antibody followed by western blotting with anti‐Flag, ‐Myc, and ‐HA antibodies. (J) Hela cells transfected with HA‐GSK3β, HA‐GSK3β (S9A), PPP1R14C or PPP1R14C (T73A) along with Myc‐TRIM25 and Flag‐Ub were subjected to IP assays with an anti‐HA antibody followed by western blotting with anti‐Flag, ‐Myc and ‐HA antibodies.

Interestingly, we found that overexpression of PPP1R14C, leading to a high level of p‐PPP1R14C (T73), increased the level of p‐GSK3β (Ser9), but reduced the level of total GSK3β. Silencing PPP1R14C or transducing the PPP1R14C (T73A) mutant had the opposite effect (Figure 7A and Figure S5A). Similar results were also observed in the mouse 4T1 cells (Figure S5B). Notably, PPP1R14C had no significant effects on GSK3β mRNA expression in TNBC cells (Figure S5C).

It has been reported that the degradation of GSK3β is mostly dependent on the proteasome pathway in lung epithelial cells.^38^ To determine whether p‐PPP1R14C regulated the GSK3β stability, we evaluated its protein half‐life period by adding cycloheximide (CHX). The half‐life of GSK3β was shortened under these treatments in PPP1R14C‐overexpressing SUM159PT and MDA‐MB‐231 cells. The reversed effect was presented in the PPP1R14C (T73A) mutant cells (Figure 7B and Figure S5D). In addition, we constructed TNBC cell lines stably expressing mutant HA‐GSK3β (S9A), which failed to be phosphorylated. IP assays were conducted in SUM159PT, SUM159PT‐HA‐GSK3β (S9A), Hela, MDA‐MB‐231, and MDA‐MA‐231‐HA‐GSK3β (S9A) cell lines to detect the ubiquitination of different GSK3β forms. These data indicated that a high level of p‐PPP1R14C (T73) promoted total and non‐phosphorylated GSK3β (S9A) ubiquitination, while the PPP1R14C (T73A) mutant inhibited this process; however, the ubiquitination level of p‐GSK3β (Ser9) was not affected by PPP1R14C or p‐PPP1R14C (Figure 7C and Figure S5E–G).

To identify which E3 ubiquitin ligases were mediated by PPP1R14C to degrade non‐phosphorylated GSK3β, mass spectrometry (MS) was applied in SUM159PT cells. Six E3 ubiquitin ligases, including TRIM25 (tripartite motif‐containing 25), TRIM21 (tripartite motif‐containing 21), ZNF598 (zinc finger protein 598), RAD18 (RAD18 E3 ubiquitin protein ligase), SH3RF3 (SH3 domain containing ring finger 3), and UBR5 (ubiquitin protein ligase E3 component N‐recognin 5), were found in MS analysis (Figure S6A). Notably, the IP assays validated that PPP1R14C interacted with ZFP598 and TRIM25 (Figure 7D). However, silencing of TRIM25, but not the other five E3 ligases increased GSK3β (Figure S6B and S6C). These observations indicated that TRIM25 might be responsible for PPP1R14C‐mediated degradation of GSK3β. Indeed, IP assays using an anti‐Flag antibody revealed that PPP1R14C formed a complex with TRIM25 depending on the phosphorylation status of PPP1R14C‐T73 (Figure 7E). In addition, an IP assay using an anti‐HA antibody was performed in Hela cells transfected with Flag‐PPP1R14C, Myc‐TRIM25, HA‐GSK3β, or HA‐GSK3β (S9A). The results confirmed that PPP1R14C mediated the interaction between TRIM25 and total/non‐phosphorylated GSK3β (Figure 7F). To identify the functional domains of the GSK3β–TRIM25 interaction, a range of truncation mutants of GSK3β and TRIM25 were generated, and were applied to perform IP assays. The results revealed that TRIM25 could only bind to those GSK3β truncations with the N1 domain, and the N1 mutant with S9A was validated in the TRIM25‐Myc immunoprecipitate, illustrating that the N‐terminal domain was essential for the GSK3β–TRIM25 interaction (Figure S6D). Furthermore, four TRIM25 truncation mutants, containing RING, B‐box, coiled‐coil (CC), and PRY/SPRY domains, were used to perform IP assays. The results demonstrated that only the TRIM25 truncation mutant containing the B‐box domain was necessary for its interaction with GSK3β (Figure S6D). As a ubiquitin E3 ligase, it was probable for TRIM25 to mediate the ubiquitination and degradation of GSK3β. To verify this hypothesis, co‐IP with an anti‐HA antibody was conducted in Hela cells transfected with Flag‐Ub, Myc‐TRIM25, HA‐GSK3β, or HA‐GSK3β (S9A). As shown in Figure 7G, TRIM25 promoted the ubiquitination of total GSK3β and non‐phosphorylated GSK3β‐S9A. To determine the role of TRIM25 as an E3 ligase of GSK3β, a TRIM25 mutant was constructed, called TRIM25‐2EA (Glu9 and Glu10 mutated to Ala), with inactivated ubiquitination activity.^28^, ^39^ IP assays exhibited that exogenous expressing wild‐type (WT) TRIM25, rather than TRIM25‐2EA, enhanced the total GSK3β and non‐phosphorylated GSK3β‐S9A ubiquitination (Figure 7H). In addition, we mutated the ubiquitination site of GSK3β (K183) and performed an exogenous co‐IP assay.^38^, ^40^ The data showed that mutation of GSK3β (K183R) interrupted the linkage of ubiquitin and GSK3β, and prevented the interaction between TRIM25 and GSK3β (Figure 7I). Furthermore, PPP1R14C overexpression substantially increased the interplay between total/non‐phosphorylated GSK3β and TRIM25, while the PPP1R14C (T73A) mutant inhibited this interaction (Figure 7J). Therefore, these results showed that p‐PPP1R14C enhanced the degradation of non‐phosphorylated GSK3β via TRIM25‐dependent ubiquitination.

### 2.8 Blockade of PPP1R14C phosphorylation inhibits TNBC progression in vitro and in vivo

**FIGURE 8:**
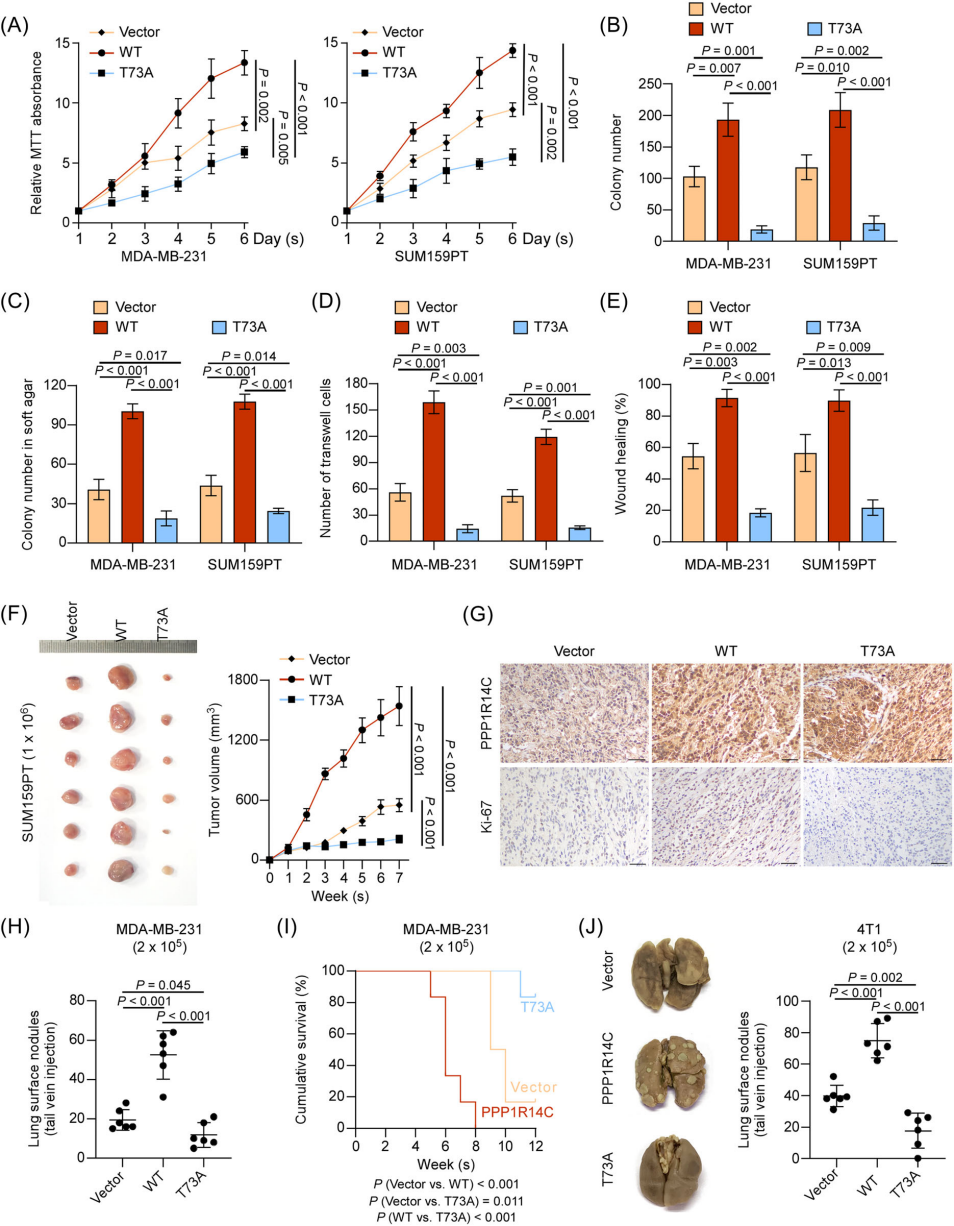
Blockade of PPP1R14C phosphorylation inhibited triple‐negative breast cancer (TNBC) progression in vitro and in vivo. (A–E) MTT (A), colony formation (B), anchorage‐independent growth (C), transwell (D), and wound healing (E) assays were performed in vector, PPP1R14C‐wild‐type (WT), or PPP1R14C (T73A) mutant in MDA‐MB‐231 and SUM159PT cells. Data represent the means ± S.D. of three independent experiments. A two‐tailed Student's *t*‐test was used. (F) SUM159PT cells transducing with WT or T73A plasmids were orthotopically injected into mice (1 × 10^6^/injection, *n* = 6/group). The tumour volumes in each group were shown. (G) IHC of PPP1R14C and Ki‐67 staining showed in the indicated xenografts. Scale bars represent 50 μm. (H) In vivo metastasis assays of vector, WT and T73A groups. The visible surface metastatic lesions were counted. (I) Kaplan–Meier survival curves of mice. (J) The visible surface metastatic lesions of the lungs were counted. Two‐tailed Student's *t*‐test and log‐rank test were used.

We established PPP1R14C‐wild type (WT) and PPP1R14C mutant in human and mouse TNBC cells. As expected, transduction with the PPP1R14C mutant impaired cell growth, invasion, G1‐S transformation, and anchorage‐independent growth of TNBC cells, suggesting that p‐PPP1R14C was essential for TNBC aggressiveness (Figure 8A–E and Figure S7A–E). Next, SUM159PT cells stably overexpressed PPP1R14C‐WT or PPP1R14C mutant, and vector cells were orthotopically injected into the mammary fat pads of mice. Tumours initiated from cells expressing the PPP1R14C mutant were much smaller than those from the vector and WT cells (Figure 8F and Figure S7F). IHC of Ki‐67 displayed that blockade of PPP1R14C phosphorylation reduced the Ki‐67 staining intensity (Figure 8G and Figure S7G). Furthermore, expressing the PPP1R14C mutant decreased the lung metastatic burden and mouse death in MDA‐MB‐231 and 4T1 cells (Figure 8H–J and Figure S7H). Collectively, these above results uncovered the fact that inhibiting PPP1R14C phosphorylation could be therapeutic against TNBC.

### 2.9 Clinical relevance of PRKCI/p‐PPP1R14C/p‐GSK3β axis in TNBC

**FIGURE 9:**
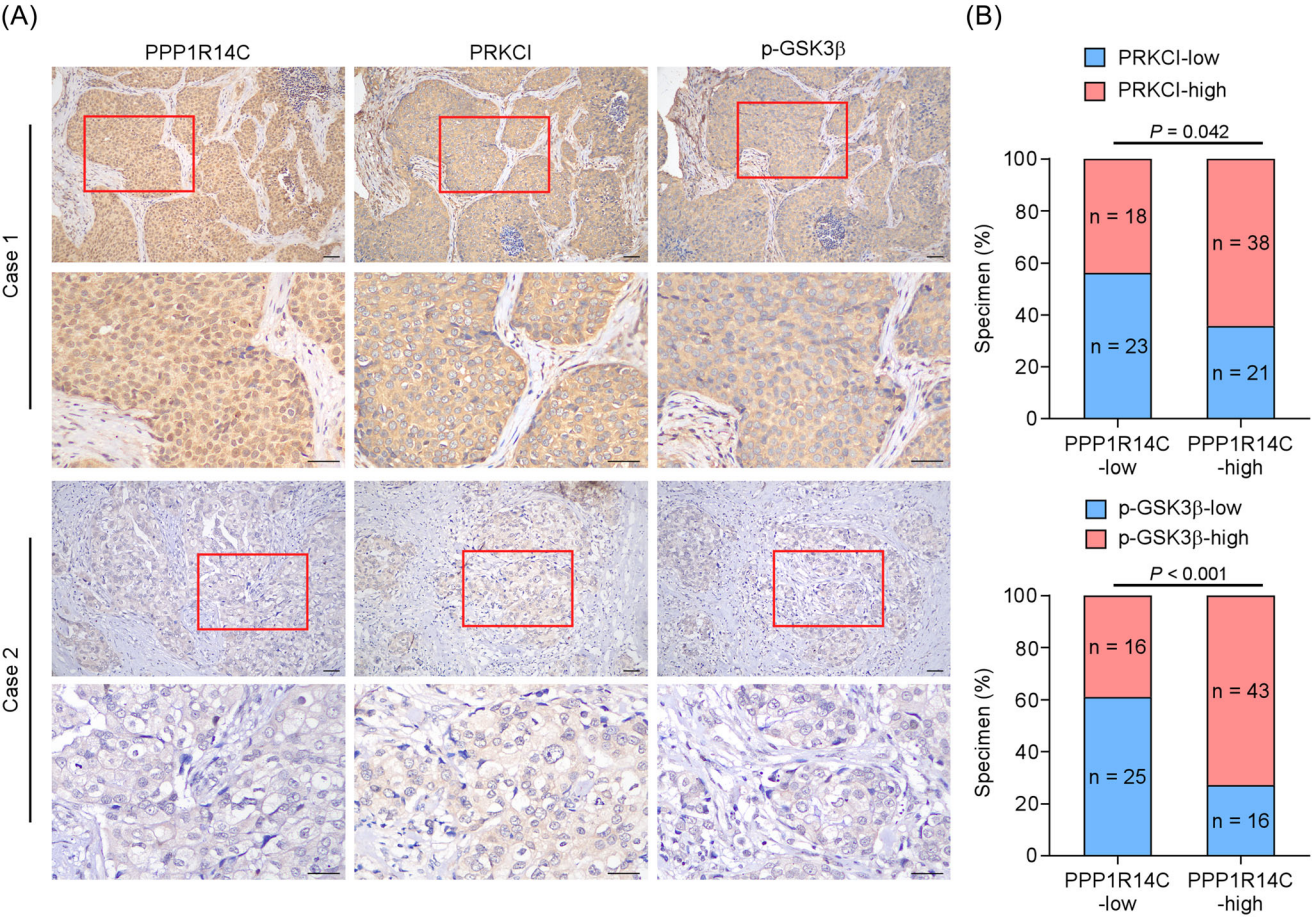
Clinical relevance of the PRKCI/p‐PPP1R14C/p‐GSK3β axis in triple‐negative breast cancer (TNBC). (A) The representative images of PRKCI, PPP1R14C and p‐GSK3β (Ser9) IHC staining in 100 specimens from patients with breast cancer. Scale bars represent 50 μm. (B) The correlation analysis revealed that PPP1R14C expression was significantly associated with PRKCI or p‐GSK3β (Ser9) expression in patient specimens (χ^2^‐test)

Finally, we assessed the PRKCI/p‐PPP1R14C/p‐GSK3β axis in clinical samples. IHC detection was performed to determine PRKCI and p‐GSK3β expression in the 100 specimens from the same cohort of the 100 TNBC patients. PPP1R14C expression correlated strongly with PRKCI and p‐GSK3β (Ser9) levels, suggesting that this axis was clinically relevant (Figure 9A,B).

## 3 DISCUSSION

The high risk of recurrence and limited therapeutic options always lead to the worst clinical outcome in TNBC patients than other subtypes of breast cancer. Therefore, effective and specific regulators are urgently needed in TNBC because of its aggressive behaviours and high metastatic potential.^41^, ^42^ Our study illustrated that PPP1R14C was overexpressed in TNBC samples and cell lines. Patients with high PPP1R14C expression were more likely to suffer shorter 5‐year OS and RFS. Furthermore, we revealed that p‐PPP1R14C (T73), which is phosphorylated by PRKCI, inactivated PP1 to sustain GSK3β phosphorylation at Ser9 via forming a complex. In addition, p‐PPP1R14C recruited TRIM25 to facilitate the ubiquitylation and degradation of non‐phosphorylated GSK3β. Moreover, blockade of PPP1R14C phosphorylation inhibited the xenograft tumorigenesis and lung metastasis of TNBC cells. The above data uncover that PPP1R14C plays an oncogenic role in TNBC and it might be a promising biomarker or target.

Lacking ER and HER‐2, TNBC does not benefit from endocrine or HER‐2 targeting therapies. Notably, recent studies indicated that TNBC was higher in immunogenicity, lymphocytes infiltration, and programmed cell death ligand 1 (PD‐L1) expression, showing sensitivity to immune checkpoint blockade therapy.^43^, ^44^ Importantly, FDA approved the use of PD‐L1 monoclonal antibody pembrolizumab for high‐risk, early‐stage TNBC patients, suggesting that immunotherapy might be a promising strategy against TNBC.^45^, ^46^ Notably, GSK3β was found to promote the phosphorylation and rapid degradation of the PD‐L1 protein.^47^ In this study, we showed that upregulation of PPP1R14C sustained the inactivation of GSK3β in TNBC. Thus, it might be postulated that PPP1R14C could promote the stability of PD‐L1 by inhibiting GSK3β. However, this hypothesis remains to be further investigated in the future, which may provide a novel regulatory mechanism for PD‐L1 expression, and suggest PPP1R14C as a potential target for immunotherapy against TNBC.

GSK3β is implicated in many cell processes, including TFs, cell‐cycle progression, cell survival, apoptosis, and migration.^48^ Non‐phosphorylated GSK3β is highly active under normal conditions and exerts an inhibitory effect on its downstream pathways.^49^ Persistently inactive GSK3β has been found in various cancers, including TNBC, suggesting that p‐GSK3β at Ser9 has an oncogenic role in neoplastic disease.^17^, ^19^ However, Cao et al. found that the active form of GSK3β might function as an oncogene, as it promoted cell proliferation by inducing S phase entry in ovarian cancer cells.^50^ The results of this study were contradictory with others, indicating that the function of GSK3β might differ in various cell types and cellular contexts.^48^ Our present data revealed that PPP1R14C, together with PRKCI, and PP1, maintained the phosphorylation of GSK3β at Ser9, which accelerated tumour proliferation and metastasis in TNBC. Furthermore, PPP1R14C promoted non‐phosphorylated GSK3β ubiquitin‐dependent degradation, which synergistically amplified and prolonged p‐GSK3β signalling in TNBC. These results present a novel mechanism to explain how GSK3β remains inactive in TNBC, and further support the oncogenic function of p‐GSK3β.

Studies have indicated that PPP1R14C regulates the protein activity depending on its inhibitory effect on PP1, and thus modulates the biological activities of numerous key proteins.^20^, ^27^, ^34^ Dedinszki et al. reported that PPP1R14C overexpression upregulated the level of phosphorylated‐RB1 and decreased the sensitivity of chemotherapy in leukaemia cells.^23^ However, studies on PPP1R14C are limited. The above findings revealed that PPP1R14C was upregulated particularly in TNBC, and the overexpression of PPP1R14C strongly correlated with the worse prognosis of patients with TNBC. Furthermore, we found that upregulating PPP1R14C, leading to a high level of p‐PPP1R14C (T73), enhanced the aggressive phenotypes of TNBC cells by sustaining the phosphorylation state of GSK3β, and facilitating the degradation of non‐phosphorylated GSK3β. The present data demonstrate that PPP1R14C might serve as a novel oncogenic biomarker, and a therapeutic target in TNBC. Although some studies reported that PPP1R14C was down‐regulated in some breast cancer cells, we discovered that PPP1R14C was detectable in all breast cancer subtypes and was specifically overexpressed in TNBC, which was supported by both GEO and TCGA datasets.^51^ These evidences suggest that PPP1R14C plays a specific role in TNBC progression.

PPP1R14C, as a PP1 inhibitory protein, shares an N‐terminal binding domain (residues 20–24; RVFFQ) with many PP1 regulatory proteins.^52^ The interaction between various PP1 regulatory proteins and PP1 is located in distinct sub‐cellular components with specific substrates.^32^, ^53^ The myosin‐targeting subunit M110 catalysed PP1 to enhance its activity toward myosin P‐light chains.^54^ In TNBC, excessive PPP1R14C could efficiently compete for PP1 binding, and disrupt PP1's interaction with its other regulatory partners. In this study, we found that the interaction between PPP1R14C and PP1 increased the specificity and affinity of PP1 for p‐GSK3β (Ser9), and strongly inhibited its dephosphorylation. In addition, PPP1R14C increased the inhibitory potency on PP1 after phosphorylation by PRKCI, which was specifically upregulated in TNBC among the PKC family members. These results prompt us to conclude that PPP1R14C‐induced persistence of p‐GSK3β specifically occurs in TNBC.

In summary, we identified an oncogenic role of PPP1R14C after phosphorylation by PRKCI in TNBC. p‐PPP1R14C inactivated PP1 to maintain the phosphorylation of GSK3β at Ser9, and induced non‐phosphorylated GSK3β ubiquitylation and degradation, contributing to the aggressive phenotype of TNBC. These findings highlight the regulatory mechanisms of GSK3β activity in TNBC, and provide new clues for therapeutic strategies. Further investigation is warranted to determine whether blockade of PPP1R14C phosphorylation might be an effective approach to the TNBC treatment.

## 4 METHODS AND MATERIALS

### 4.1 Cell lines

MCF‐7, ZR75‐30, SK‐BR‐3, HCC1937, MDA‐MB‐231, BT‐549, 4T1, and Hela were from the American Type Culture Collection (ATCC) and SUM159PT was supplied by Asterand Bioscience. Roswell Park Memorial Institute (RPMI) 1640 with 10% fetal bovine serum (FBS) was used to culture BT‐549, and Ham's F‐12 with 5% FBS was applied to culture SUM159PT. Other cell lines were cultured in Dulbecco's modified Eagle medium (DMEM) with 5% FBS. Cells were maintained at 37°C in an incubator with 5% CO_2_. In the cell culture, the MycoSEQ™ Mycoplasma Detection Kit (#4460623, Thermo Fisher Scientific) was used to identify mycoplasma contamination. CHX (ApexBio) at 50 μg/ml was added to suppress translation. The cell lines included in our study were qualified by a short tandem repeat (STR) profiling test at the beginning of our experiments.

### 4.2 Clinicopathological characteristics of patients

Retrospectively, we overviewed the electronic medical record data of Sun Yat‐sen University Cancer Center from 2004 to 2012. One hundred fifty patients who were histopathologically diagnosed with breast cancer with surveillance data were included in our study. One hundred fifty archived breast cancer samples and 10 tumour‐adjacent tissues were further analysed. Detailed clinicopathological data and survival data were collected (Table S2). The study was authorized by the ethics committee of Sun Yat‐sen University Cancer Center.

### 4.3 Quantitative real‐time PCR

The RNA was extracted by using TRIzol kit (Thermo Fisher Scientific). Two microgram RNA was mixed with RNase‐free DNase, and then reversely transcribed into cDNA. One microliter of cDNA was used to conduct qPCR in SYBR premix Ex Taq (Takara) on the CFX96 Real‐Time PCR Detection System (Bio‐Rad). The Ct value of target genes was compared to that of glyceraldehyde‐3‐phosphate dehydrogenase (GAPDH) to define the relative expression level.

### 4.4 Western blotting

The cellular proteins were extracted by Radioimmunoprecipitation assay lysis buffer (Thermo Fisher Scientific). The concentration of prepared protein was identified through a Bio‐Rad DC protein assay kit II. The same amount of protein lysate samples was loaded separately into 8% to 15% SDS‐PAGE gels, and samples were segregated by electrophoresis, and then the separated proteins were transferred onto polyvinylidene fluoride (PVDF) transfer membranes (Thermo Fisher Scientific). The membranes were blocked with 5% defatted milk, and then were treated with the primary antibodies overnight at 4°C, and subsequently were treated with labelled secondary antibodies. The immunoreactive proteins were visualized by ECL chemiluminescent substrate reagent kit (Thermo Fisher Scientific). Detailed information on the antibodies used was listed in Table S5.

### 4.5 Immunohistochemistry

IHC was conducted to determine PPP1R14C in 10 matched tumour‐adjacent tissues and 150 breast cancer specimens using an anti‐PPP1R14C (#PA5‐50996, Invitrogen) antibody. To calculate the SI, the intensity and proportion of positively stained tumour cells were counted. The intensity was catalogued into three levels: no staining was defined as 0; weak staining was defined as 1; moderate staining was defined as 2; strong staining was defined as 3. The positively‐stained proportion was classified into four scores: negative was considered as 0; lower than 10% was considered as 1; 10% to 35% was considered as 2; 35% to 75% was considered as 3; 75% to 100% was considered as 4. The rank of the staining score was determined by using SI. SI consisted of 9 scores: 0, 1, 2, 3, 4, 6, 8, 9, and 12. The cutoff value of PPP1R14C was SI = 6; SI equal to or higher than 6 was defined as high expression, and SI lower than 6 was defined as low, respectively. The threshold was identified by the heterogeneity of the log‐rank test of 5‐year OS and RFS.

### 4.6 Generation of stably transfected cell lines

Transient plasmid transfection was accomplished by using Lipofectamine 3000 (Thermo Fisher Scientific). To construct the stable PPP1R14C‐knockdown cells, human cancer cell lines, MDA‐MB‐231 and SUM159PT, and a mouse‐derived TNBC cell line 4T1 were infected with a retrovirus plasmid containing two different short hairpin RNAs (human and mouse) targeting PPP1R14C, respectively (GeneChem, Shanghai, China). To establish the PPP1R14C‐overexpressing cell line, the full‐length sequence of PPP1R14C cDNA was identified and cloned to generate a Flag‐PPP1R14C construct. The construct was packaged into lentiviruses, which further infected the MDA‐MB‐231, SUM159PT, and 4T1 cells to integrate PPP1R14C gene into the host cell genome. The positive clones were selected by using puromycin. The constructing primer sequences were listed in Table S6.

### 4.7 Xenograft tumour models

The animal experiments were all admitted by Sun Yat‐sen University's Institutional Animal Care and Use Committee. In brief, five to six‐week‐old female BALB/c‐nu mice (18–20 g in weight) were provided by Guangdong Medical Laboratory Animal Center. Mice were raised in the SPF‐levelled barrier system in the Laboratory Animal Center of the Sun Yat‐sen University. To generate the orthotopic xenograft model and spontaneous metastasis models, a small incision between the fourth nipple and the midline of mice was made to expose the mammary fat pad. Fifty microliter of cell suspension (SUM159PT, 1 × 10^6^; 4T1, 2 × 10^5^, respectively) in an insulin syringe was then injected into the mammary fat pad and the incision was sutured. Tumour volumes were determined every week after the surgery. The spontaneous metastasis of 4T1 cells was evaluated using the Xenogen IVIS Spectrum Imaging System (Caliper Life Sciences).

For lung colonization models, mice were randomly divided into groups (six mice/group), treated under intravenous injection with 2 × 10^5^ MDA‐MB‐231 cells. BLI was employed periodically to monitor the lung metastatic lesions and the spontaneously metastatic lesions through the Xenogen IVIS Spectrum Imaging System.^55^ Twelve weeks later, the lungs were removed after execution of the mice, fixed in formalin, and embedded in paraffin. The amount of lung metastatic burdens was counted by five random fields under low magnification, and the data were presented in the form of mean ± S.D.

### 4.8 PP1 activity assay by using colorimetric methods

The activity of PP1 was measured according to the manufacturer's instructions (GENMED SCIENTIFICS INC., USA). 5 × 10^6^ cells were prepared for the measurement. Samples were washed with GENMED clearing buffer and lysed with GENMED lysis buffer. Five tubes of standardized buffer (standard concentration of phosphate) were prepared in the measurement system, and the standard curve was established. After the assay of the sample background, we detected the sample activity and obtained the corresponding concentration of phosphate according to the standard curve, then calculated the sample activity. The enzyme activity was measured based on the determination of free phosphate with the colour development. Absorbance was read at 660 nm.

### 4.9 Statistical analysis

Statistical analysis was conducted by using the SPSS software (version 21.0, IBM). Continuous variables with the normal distribution were compared by a two‐tailed Student's *t*‐test. Qualitative variables and non‐normally distributed continuous variables were analysed by the Mann–Whitney *U*‐test or Chi‐square test. Kaplan–Meier analysis was used for univariate survival analysis and the log‐rank test was applied to compare different survival curves. For the multivariate analysis, Cox regression analysis was applied. A *P*‐value lower than 0.05 at two sides was deemed statistically significant.

## CONFLICT OF INTEREST

The authors declare that there is no conflict of interest that could be perceived as prejudicing the impartiality of the research reported.

## Supporting information

Figure S1

Figure S2

Figure S3

Figure S4

Figure S5

Figure S6

Figure S7

Tables S1‐S6
