# Supplementary figures and images for: Protein phosphatase 1 regulatory inhibitor subunit 14C promotes triple‐negative breast cancer progression via sustaining inactive glycogen synthase kinase 3 beta
Source: Clin Transl Med. 2022 Jan 28;12(1):e725. doi: 10.1002/ctm2.725 (PMC8797469; doi:10.1002/ctm2.725)

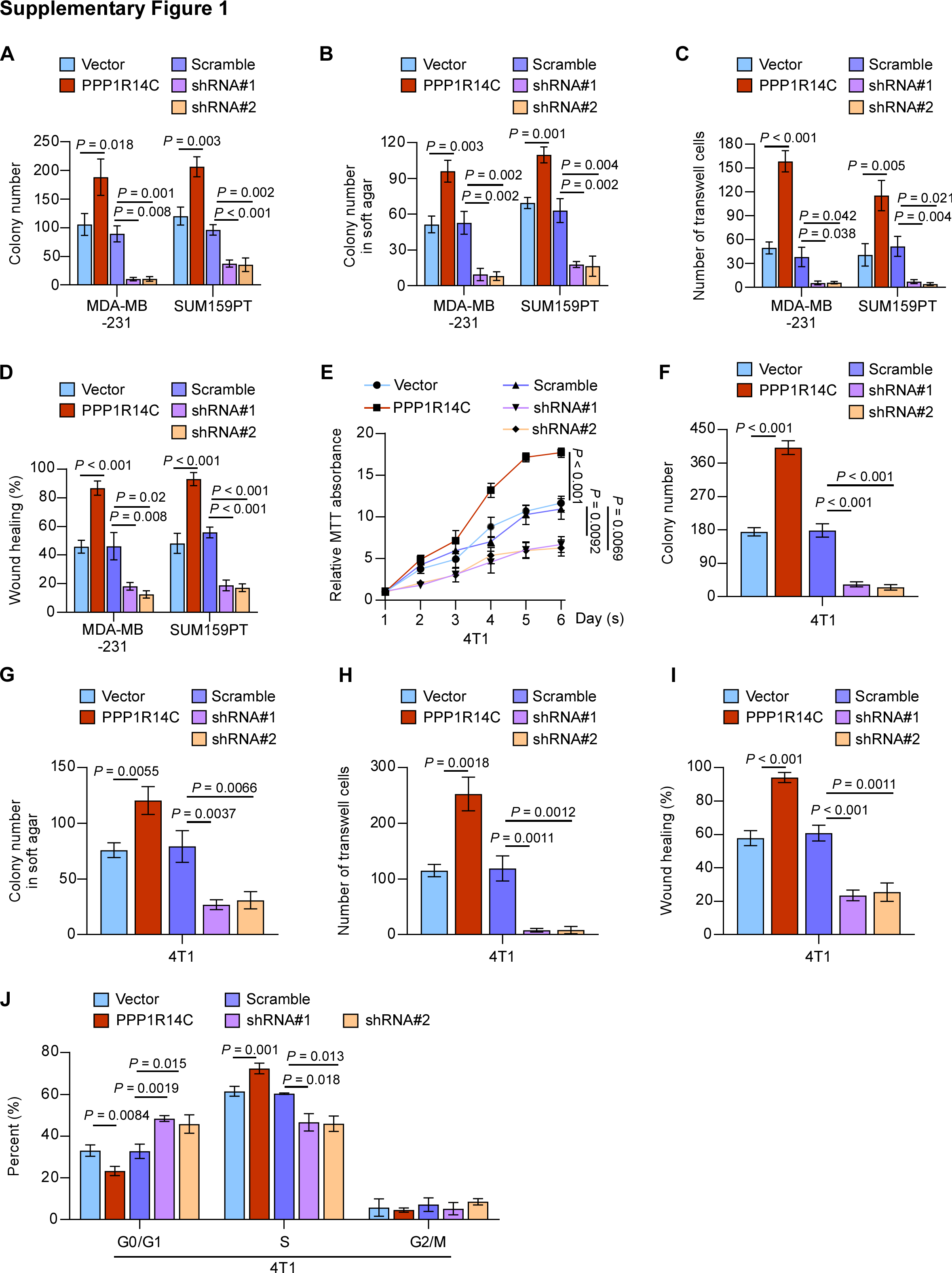

Supplement: Supplementary file 1 — Figure S1 [file CTM2-12-e725-s001.tif]

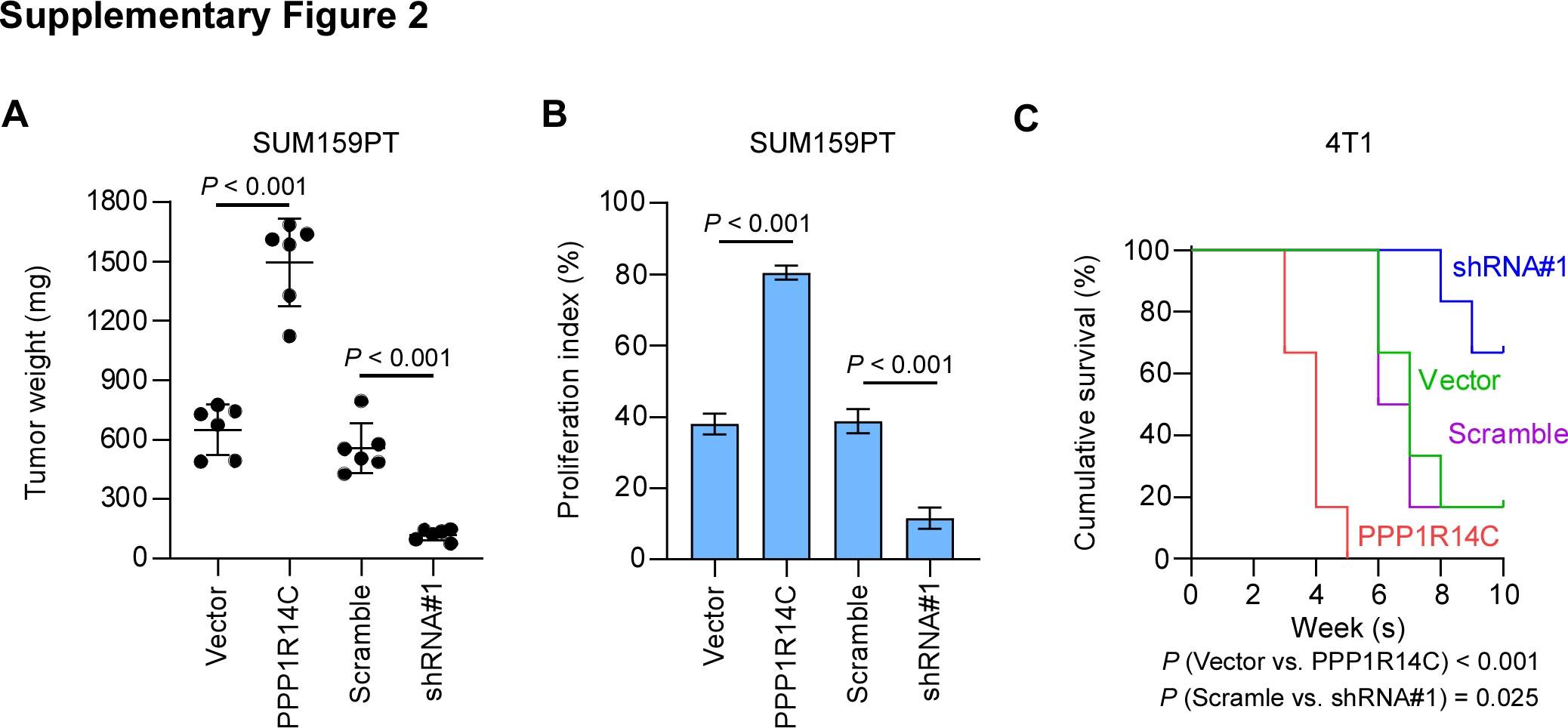

Supplement: Supplementary file 2 — Figure S2 [file CTM2-12-e725-s008.tif]

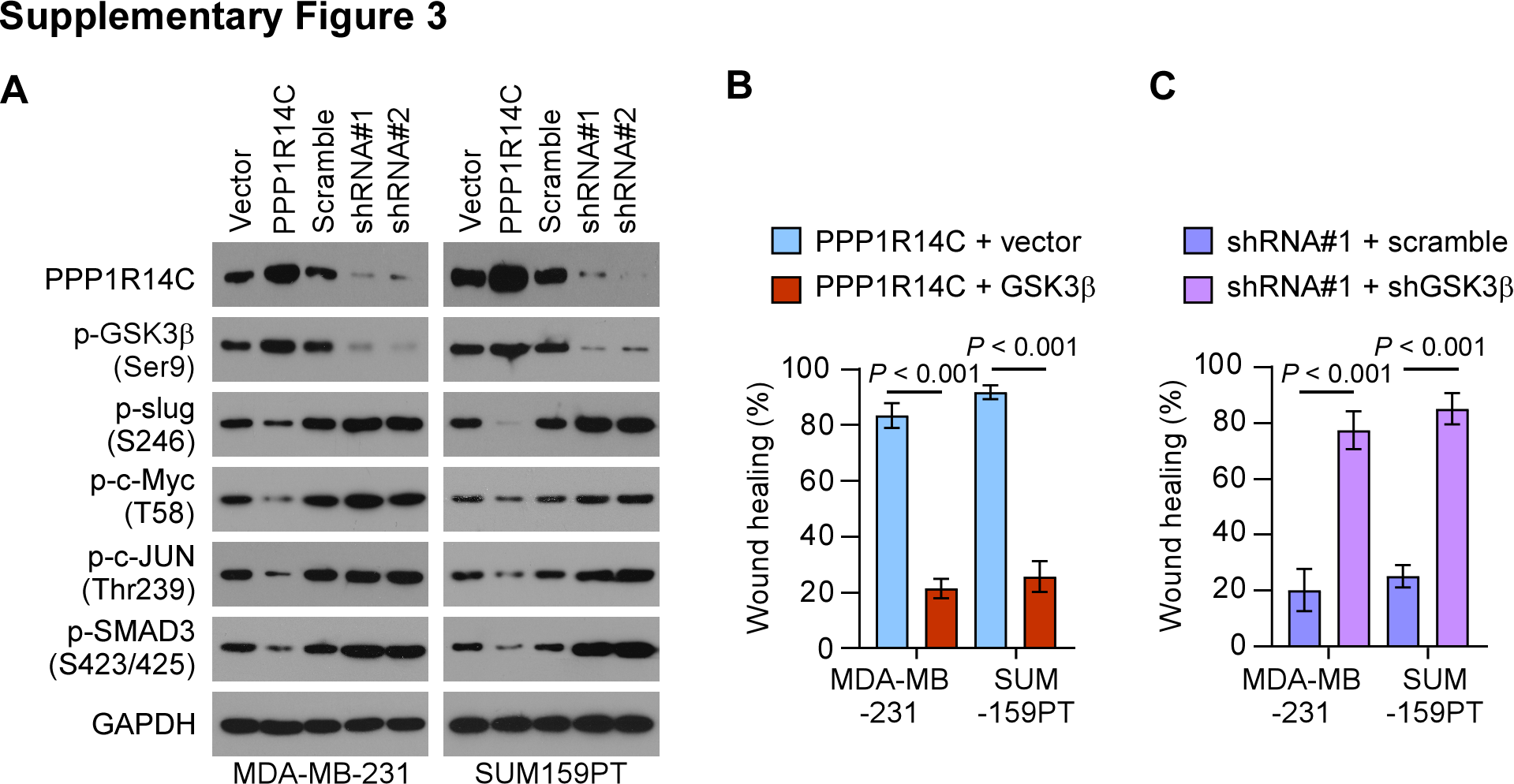

Supplement: Supplementary file 3 — Figure S3 [file CTM2-12-e725-s004.tif]

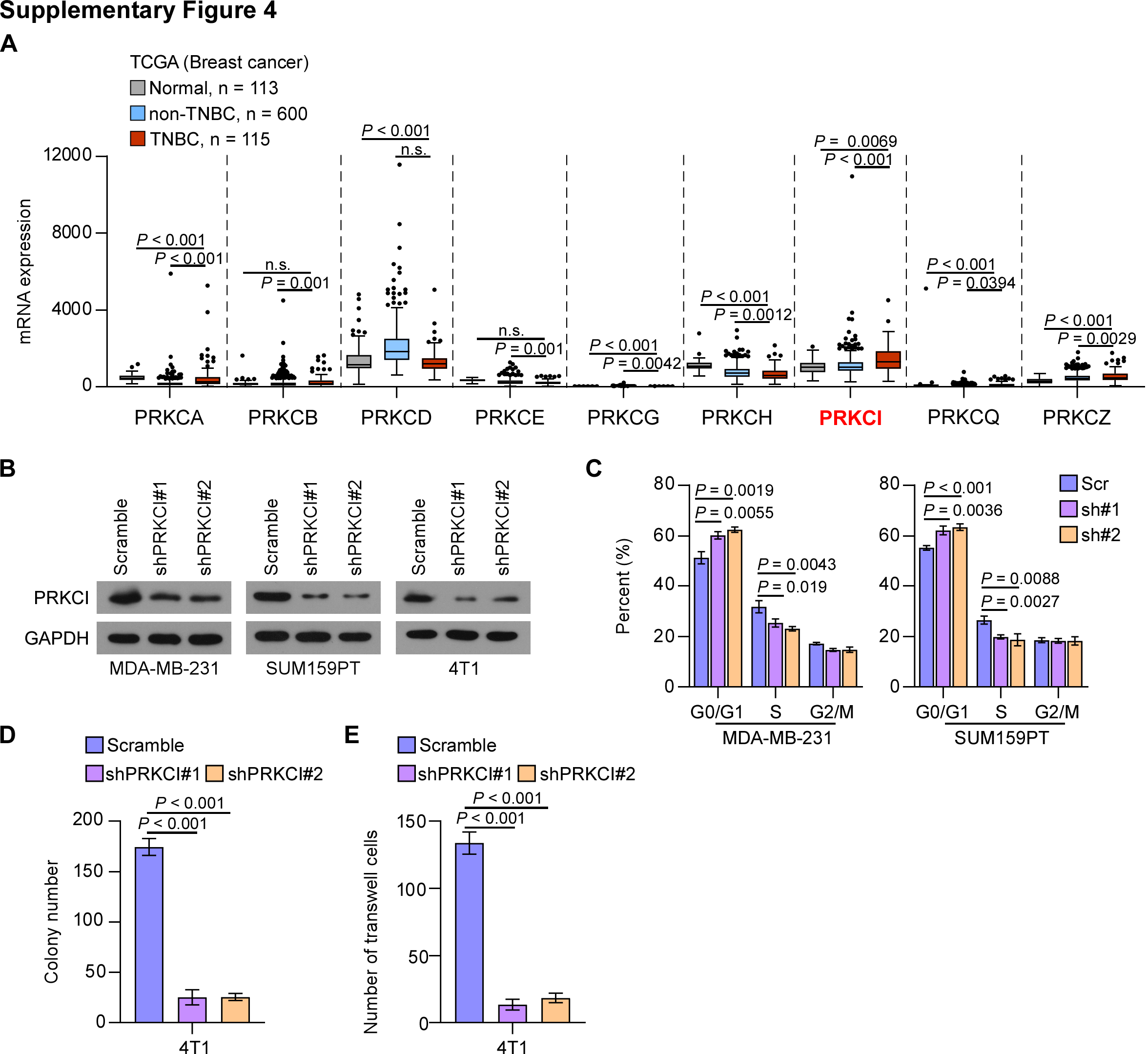

Supplement: Supplementary file 4 — Figure S4 [file CTM2-12-e725-s003.tif]

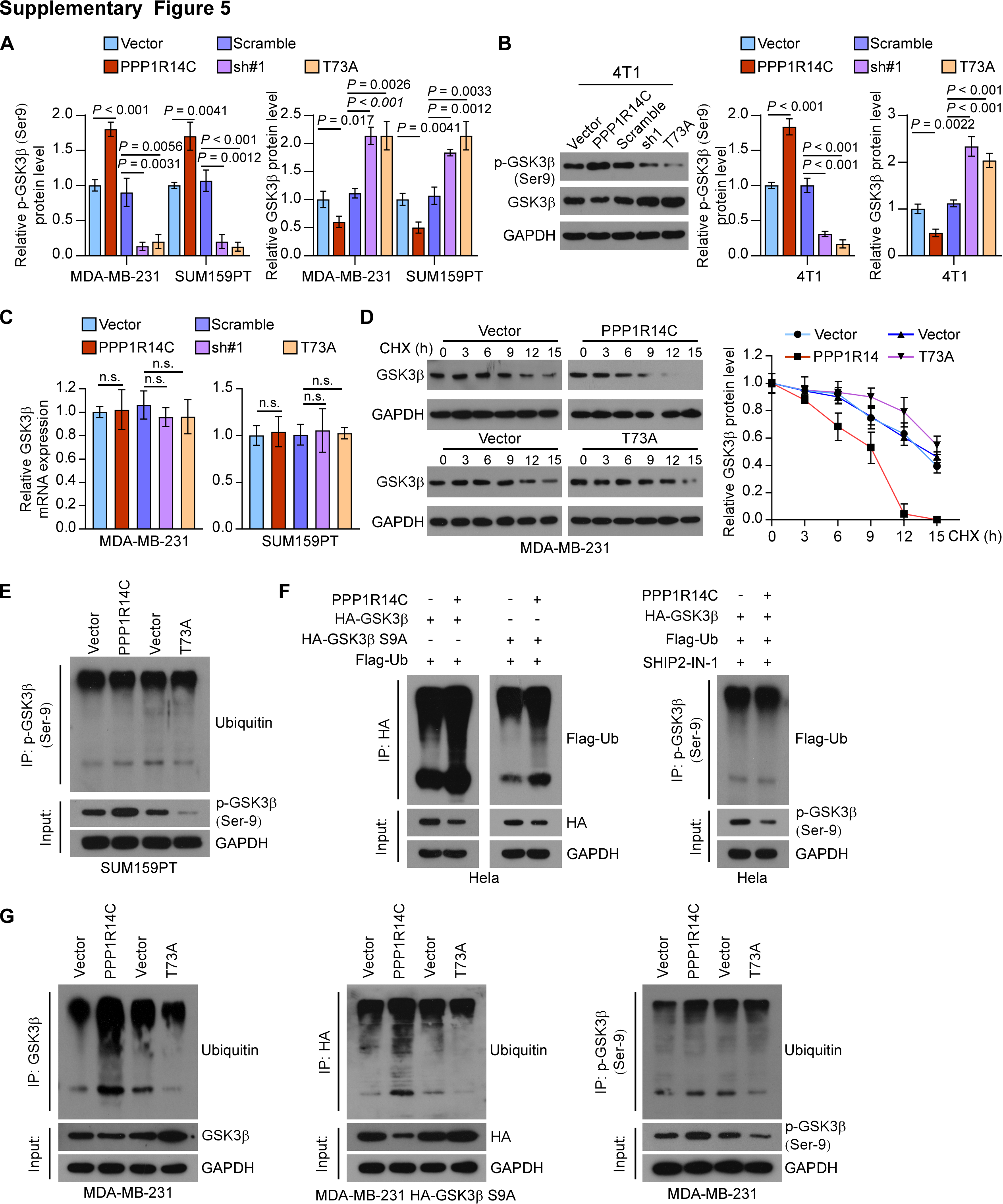

Supplement: Supplementary file 5 — Figure S5 [file CTM2-12-e725-s005.tif]

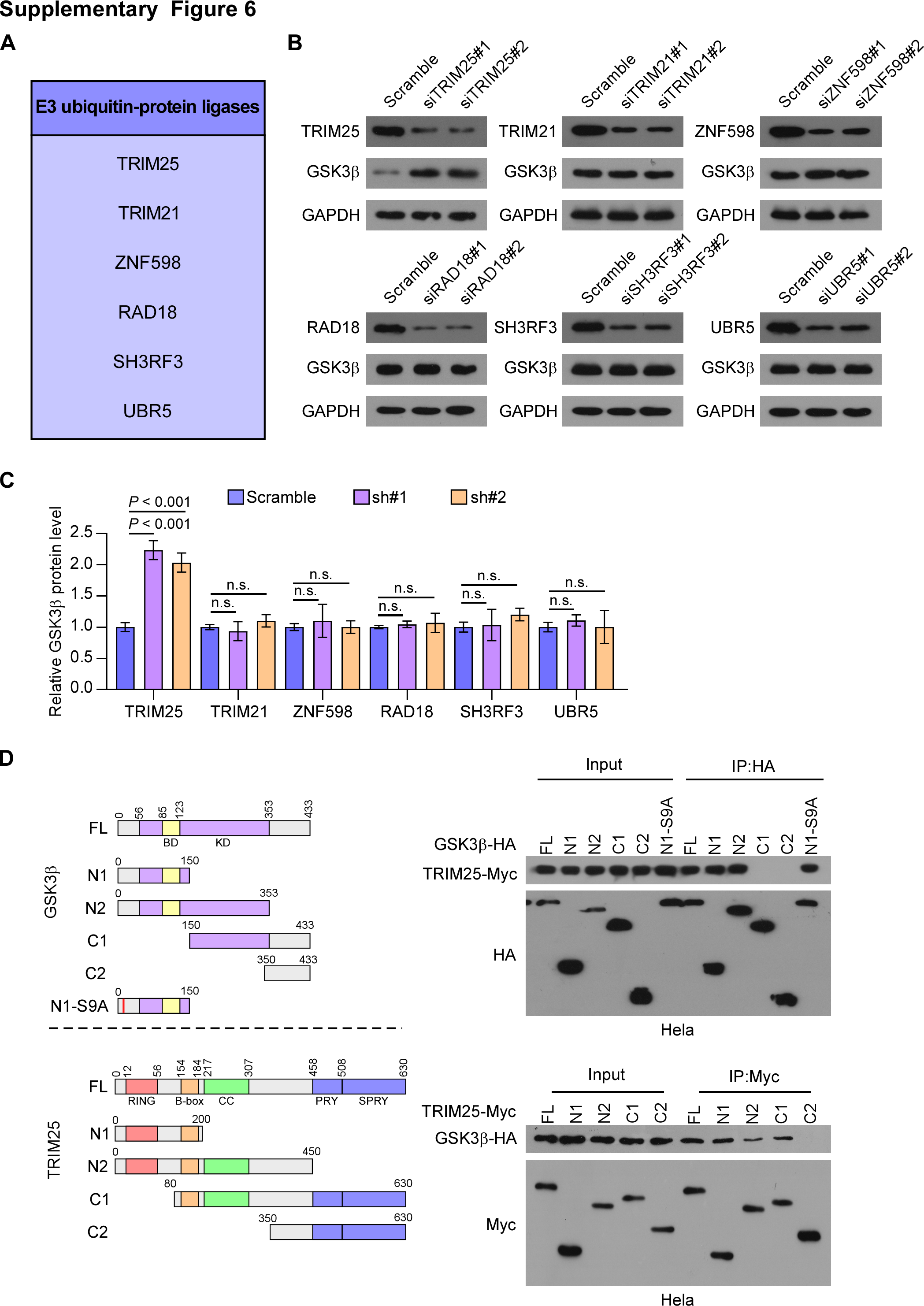

Supplement: Supplementary file 6 — Figure S6 [file CTM2-12-e725-s006.tif]

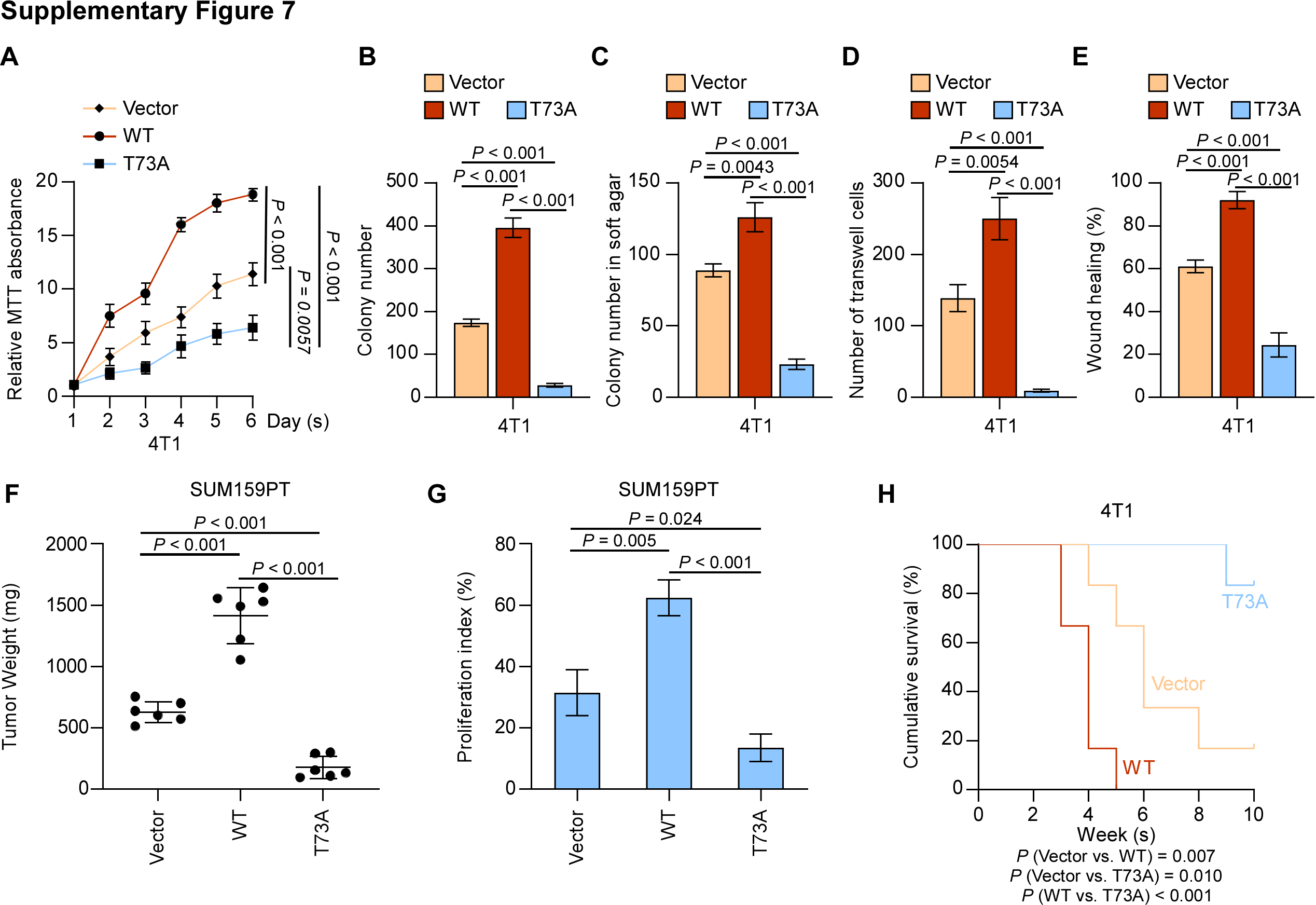

Supplement: Supplementary file 7 — Figure S7 [file CTM2-12-e725-s002.tif]
