# Supplementary material for: Protein phosphatase 1 regulatory inhibitor subunit 14C promotes triple‐negative breast cancer progression via sustaining inactive glycogen synthase kinase 3 beta
Source: Clin Transl Med. 2022 Jan 28;12(1):e725. doi: 10.1002/ctm2.725 (PMC8797469; doi:10.1002/ctm2.725)
Supplement: Supplementary file 8 — Tables S1‐S6 [file CTM2-12-e725-s007.docx]

**Table S1. 48 genes in Figure 1B**

| **Name** | **Gene ID** | **Description** |
| --- | --- | --- |
| KRT16 | 3868 | Keratin 16 [Homo sapiens] |
| BPI | 671 | Bactericidal permeability increasing protein [Homo sapiens] |
| SHC4 | 399694 | SHC adaptor protein 4 [Homo sapiens] |
| ABCA13 | 154664 | ATP binding cassette subfamily A member 13 [Homo sapiens] |
| MAP7D2 | 256714 | MAP7 domain containing 2 [Homo sapiens] |
| RASAL1 | 8437 | RAS protein activator like 1 [Homo sapiens] |
| FOXC1 | 2296 | Forkhead box C1 [Homo sapiens] |
| FOSL1 | 8061 | FOS like 1, AP-1 transcription factor subunit [Homo sapiens] |
| VXN | 254778 | Vexin [Homo sapiens] |
| RHCG | 51458 | Rh family C glycoprotein [Homo sapiens] |
| LOC84856 | 84856 | Long intergenic non-protein coding RNA 839 [Homo sapiens] |
| MAGEB4 | 4115 | MAGE family member B4 [Homo sapiens] |
| COL11A2 | 1302 | Collagen type XI alpha 2 chain [Homo sapiens] |
| CHI3L1 | 1116 | Chitinase 3 like 1 [Homo sapiens] |
| PPP1R14C | 81706 | Protein phosphatase 1 regulatory inhibitor subunit 14C [Homo sapiens] |
| CCL18 | 6362 | C-C motif chemokine ligand 18 [Homo sapiens] |
| CHI3L2 | 1117 | Chitinase 3 like 2 [Homo sapiens] |
| VGLL1 | 51442 | Vestigial like family member 1 [Homo sapiens] |
| UGT8 | 7368 | UDP glycosyltransferase 8 [Homo sapiens] |
| SFRS13B | 135295 | Serine and arginine rich splicing factor 12 [Homo sapiens] |
| CHRM3 | 1131 | Cholinergic receptor muscarinic 3 [Homo sapiens] |
| KRT83 | 3889 | Keratin 83 [Homo sapiens] |
| GJB3 | 2707 | Gap junction protein beta 3 [Homo sapiens] |
| BCL11A | 53335 | BAF chromatin remodeling complex subunit BCL11A [Homo sapiens] |
| CYP27C1 | 339761 | Cytochrome P450 family 27 subfamily C member 1 [Homo sapiens] |
| GFRA3 | 2676 | GDNF family receptor alpha 3 [Homo sapiens] |
| LEMD1 | 93273 | LEM domain containing 1 [Homo sapiens] |
| TAFA3 | 284467 | TAFA chemokine like family member 3 [Homo sapiens] |
| PADI2 | 11240 | Peptidyl arginine deiminase 2 [Homo sapiens] |
| OPRK1 | 4986 | Opioid receptor kappa 1 [Homo sapiens] |
| AMY1A | 276 | Amylase alpha 1A [Homo sapiens] |
| CHST4 | 10164 | Carbohydrate sulfotransferase 4 [Homo sapiens] |
| ART3 | 419 | ADP-ribosyltransferase 3 [Homo sapiens] |
| COCH | 1690 | Cochlin [Homo sapiens] |
| PSAT1 | 29968 | Phosphoserine aminotransferase 1 [Homo sapiens] |
| MMP7 | 4316 | Matrix metallopeptidase 7 [Homo sapiens] |
| WNT6 | 7475 | Wnt family member 6 [Homo sapiens] |
| KRT6C | 286887 | Keratin 6C [Homo sapiens] |
| S100A2 | 6273 | S100 calcium binding protein A2 [Homo sapiens] |
| PNMA3 | 29944 | PNMA family member 3 [Homo sapiens] |
| LCN2 | 3934 | Lipocalin 2 [Homo sapiens] |
| KRT23 | 25984 | Keratin 23 [Homo sapiens] |
| CHODL | 140578 | Chondrolectin [Homo sapiens] |
| KRT6A | 3853 | Keratin 6A [Homo sapiens] |
| PHGDH | 26227 | Phosphoglycerate dehydrogenase [Homo sapiens] |
| RGR | 5995 | Retinal G protein coupled receptor [Homo sapiens] |
| MELTF | 4241 | Melanotransferrin [Homo sapiens] |
| FZD9 | 8326 | Frizzled class receptor 9 [Homo sapiens] |

**Table S2. Clinicopathological characteristics of 150 breast cancer patients**

| **Parameters** | | | **Number of cases (%)** | |
| --- | --- | --- | --- | --- |
| **Age** | | |  | |
| < 45 | | | 54 (36.0) | |
| ≥ 45 | | | 96 (64.0) | |
| **Clinical stage** | | |  | |
| I-II | | | 106 (70.7) | |
| III-IV | | | 44 (29.3) | |
| **T classification** | | |  | |
| T1-2 | | | 126 (84.0) | |
| T3-4 | | | 24 (16.0) | |
| **N classification** | | |  | |
| N0 | | | 80 (53.3) | |
| N1-N3 | | | 70 (46.7) | |
| **M classification** | | |  | |
| M0 | | | 147 (98.0) | |
| M1 | | | 3 (2.0) | |
| **Histologic grade** | | |  | |
| G1-2 | | | 70 (46.7) | |
| G3 | | | 80 (53.3) | |
| **ER status** | | |  | |
| Negative | | | 109 (72.7) | |
| Positive | | | 41 (27.3) | |
| **PR status** | | |  | |
| Negative | | | 110 (73.3) | |
| Positive | | | 40 (26.7) | |
| **HER-2 status** | | |  | |
| Negative | 137 (91.3) | |  |  |
| Positive | | | 13 (8.7) | |
| **TNBC**  No | | | 50 (33.3) | |
| Yes | | | 100 (66.7) | |
| **Ki-67**  ≥ 14% | | | 112 (74.7) | |
| < 14% | | | 38 (25.3) | |
| **Vital status** | | |  | |
| alive | | | 122 (81.3) | |
| dead | | | 28 (18.7) | |
| **Relapse** | | |  | |
| No | | | 121 (80.7) | |
| Yes | | | 29 (19.3) | |
| **PPP1R14C expression** | | |  | |
| Low | | | 80 (53.3) | |
| High | | | 70 (46.7) | |

**Clinicopathological characteristics of 100** **triple-negative breast cancer patients**

| **Parameters** | **Number of cases (%)** |
| --- | --- |
| **Age** |  |
| < 45 | 31 (31.0) |
| ≥ 45 | 69 (69.0) |
| **Clinical stage** |  |
| I-II | 67 (67.0) |
| III-IV | 33 (33.0) |
| **T classification** |  |
| T1-2 | 84 (84.0) |
| T3-4 | 16 (16.0) |
| **N classification** |  |
| N0 | 51(51.0) |
| N1-N3 | 49 (49.0) |
| **M classification** |  |
| M0 | 97 (97.0) |
| M1 | 3 (3.0) |
| **Histologic grade** |  |
| G1-2 | 45 (45.0) |
| G3 | 55 (55.0) |
| **Ki-67** |  |
| ≥ 14% | 79 (79.0) |
| < 14% | 21 (21.0) |
| **Vital status** |  |
| alive | 80 (80.0) |
| dead | 20 (20.0) |
| **Relapse** |  |
| No | 77 (77.0) |
| Yes | 23 (23.0) |
| **PPP1R14C expression** |  |
| Low | 41 (41.0) |
| High | 59 (59.0) |

**Table S3. Correlation between PPP1R14C and clinicopathological characteristics of triple-negative breast cancer patients (n = 100)**

|  | **PPP1R14C expression** | |  |
| --- | --- | --- | --- |
| Characteristics | Low,  no. cases (%) | High,  no. cases (%) | *P* values |
| **Age**  < 45  ≥ 45 | 13 (41.94 %)  28 (40.58 %) | 18 (58.06 %)  41 (59.42 %) | 0.899 |
| **Clinical stage** |  |  |  |
| I-II | 32 (47.76 %) | 35 (52.24 %) | 0.050 |
| III-IV | 9 (27.27 %) | 24 (72.73 %) |  |
| **T** **classification** |  |  |  |
| T1-2 | 39 (46.43 %) | 45 (53.57 %) | 0.013 |
| T3-4 | 2 (12.50 %) | 14 (87.50 %) |  |
| **N classification** |  |  |  |
| N0 | 18 (35.29 %) | 33 (64.71 %) | 0.237 |
| N1-3 | 23 (46.94 %) | 26 (53.06 %) |  |
| **M classification** |  |  |  |
| M0 | 40 (41.24 %) | 57 (58.76 %) | 1.00 |
| M1 | 1 (33.33 %) | 2 (66.67 %) |  |
| **Histologic grade** |  |  |  |
| G1-2 | 17 (37.78 %) | 28 (62.22 %) | 0.553 |
| G3 | 24 (43.64 %) | 31 (56.36 %) |  |
| **Ki-67** |  |  |  |
| < 14% | 10 (47.62 %) | 11 (52.38 %) | 0.488 |
| ≥ 14% | 31 (39.24 %) | 48 (60.76 %) |  |
| **Relapse** |  |  |  |
| No | 38 (49.35 %) | 39 (50.65 %) | 0.002 |
| Yes | 3 (13.04 %) | 20 (86.96 %) |  |

**Table S4. Univariate and multivariate analysis of factors associated with 5-year overall survival in patients with TNBC (n = 100)**

| Characteristics | **Univariate analysis** | | | **Multivariate analysis** | |
| --- | --- | --- | --- | --- | --- |
|  | HR (95% CI) | *P* values | HR (95% CI) | | *P* values |
| **Age**  (≥45 vs. <45) | 0.6023 (0.229 - 1.588) | 0.261 | 0.633 (0.251 - 1.596) | | 0.332 |
| **Expression of PPP1R14C**  (high vs. low) | 5.046 (2.097 - 12.14) | 0.004 | 3.544 (1.005 - 12.493) | | 0.049 |
| **T classification**  (T3-4 vs. T1-2) | 5.225 (1.31 - 20.84) | < 0.001 | 5.060 (1.770 - 14.466) | | 0.002 |
| **N classification**  (N1-3 vs. N0) | 0.655 (2.27 - 1.57) | 0.345 | 0.797 (0.322 - 1.970) | | 0.623 |
| **Histologic grade**  (G3 vs. G1-2) | 0.720 (0.30 - 1.75) | 0.459 | 0.426 (0.156 - 1.163) | | 0.096 |
| **Ki-67**  (≥14% vs. <14%) | 1.556 (0.536 - 4.519) | 0.476 | 1.985 (0.529 - 7.446) | | 0.309 |

HR, hazard ratio; CI, confidence interval.

**Univariate and multivariate analysis of factors associated with 5-year relapse-free survival in patients with TNBC (n = 100)**

| **Characteristics** | **Univariate analysis** | | | **Multivariate analysis** | |
| --- | --- | --- | --- | --- | --- |
|  | HR (95% CI) | *P* values | HR (95% CI) | | *P* values |
| **Age**  (≥45 vs. <45) | 0.555 (0.227 - 1.354) | 0.154 | 0.618 (0.261 - 1.464) | | 0.274 |
| **Expression of PPP1R14C**  (high vs. low) | 6.164 (2.72 - 13.97) | < 0.001 | 4.614 (1.309 - 16.266) | | 0.017 |
| **T classification**  (T3-4 vs. T1-2) | 4.11 (1.41 - 14.82) | < 0.001 | 3.303 (1.230 - 8.867) | | 0.018 |
| **N classification**  (N2-3 vs. N0-1) | 0.669 (0.30 - 1.52) | 0.341 | 0.943 (0.399 - 2.230) | | 0.893 |
| **Histologic grade**  (G3 vs. G1-2) | 0.641 (0.28 - 1.47) | 0.281 | 0.441 (0.177 - 1.101) | | 0.079 |
| **Ki67**  (≥14% vs. <14%) | 1.273 (0.47 - 3.46) | 0.659 | 1.264 (0. .399 - 4.010) | | 0.690 |

HR, hazard ratio; CI, confidence interval.

**Table S5. Antibodies with the applications in which they were used**

| **Name** | **Catalog number** | **Company** | **Concentration** | **Source** |
| --- | --- | --- | --- | --- |
| anti-PPP1R14C | PA5-50996 | Invitrogen | 1:1000 | Rabbit |
| anti-PP1 | sc-7482 | Santa Cruz  Biotechnology | 1:500 | Mouse |
| anti-GSK3β | 22104-1-AP | Proteintech | 1:2000 | Rabbit |
| anti-p-GSK3β (Ser9) | #9323 | CST | 1:1000 | Rabbit |
| anti-Phospho-Threonine (42H4) | #9386 | CST | 1:1000 | Mouse |
| anti-HA | H6908 | Sigma- Aldrich | 1:1000 | Rabbit |
| anti-Flag | F7425 | Sigma- Aldrich | 1:1000 | Rabbit |
| anti-Myc | 05-724 | Sigma- Aldrich | 1:1000 | Mouse |
| anti-GAPDH | #5174 | CST | 1:1000 | Rabbit |
| anti-PRKCI | #2998 | CST | 1:1000 | Rabbit |
| and-TRIM25 | HPA005909 | Sigma | 1:1000 | Rabbit |
| anti-p-p53 (Ser15) | MA5-15229 | Invitrogen | 1:1000 | Mouse |
| anti-p-CREB (Ser133/ Ser63) | MA1-114 | Invitrogen | 1:1000 | Mouse |
| anti-p-eIF2A (Ser51) | #3398 | CST | 1:1000 | Rabbit |
| anti-p-Aurora A (Thr288) | MA5-14904 | Invitrogen | 1:1000 | Rabbit |
| anti-p-Slug (S246) | ab63568 | Abcam | 1:500 | Rabbit |
| anti-p- c-Myc (T58) | ab185655 | Abcam | 1:1000 | Rabbit |
| anti-c-Jun (Thr239) | PA5-104748 | Invitrogen | 1:1000 | Rabbit |
| anti-p-SMAD3 (S423/ 425) | #9520 | CST | 1:1000 | Rabbit |

**Table S6. Primers**

| **Primers for real-time PCR** | | **Sequence(5'-3')** | |
| --- | --- | --- | --- |
| PPP1R14C | forward | ACGATCGTAAGGAGCTTCGG | |
|  | reverse | CACGATCCATTCCTCCAGCA | |
| GSK3β | forward | ATTTCACCTCAGGAGTGCGG | |
|  | reverse | AAGAGTGCAGGTGTGTCTCG | |
| GAPDH | forward | GTCTCCTCTGACTTCAACAGCG | |
|  | reverse | ACCACCCTGTTGCTGTAGCCAA | |
| **Primers for plasmid constructs** | | | |
| pLVX-IRES-Hyg –PPP1R14C | forward | GgattcATGTCGGTGGCGACGGGCAGCAG | |
|  | reverse | AgatctTCATACACTCTTCTGCGGAGGGC | |
| **Target sequence**  pSuper-retro-neo  –PPP1R14C-shRNA#1 (Homo sapiens) |  | GCTCTTGTAGACTGCTACAAA | |
| pSuper-retro-neo –PPP1R14C-shRNA#2 |  | GCCAGAGGTAGAAATTGACAT |  |
| (Homo sapiens) |  |  |  |
| pSuper-retro-neo –Ppp1r14c-shRNA#1 |  | GCTTCGAAATTACAGGAAGCT |  |
| (Mus musculus) |  |  |  |
| pSuper-retro-neo –Ppp1r14c -shRNA#2  (Mus musculus) |  | GAAGAAGAAATGCCAGATGTA |  |
